# Isothermal amplification and colorimetric detection of *Vibrio cholerae* in environmental matrices

**DOI:** 10.1128/spectrum.02753-25

**Published:** 2026-05-21

**Authors:** M. Adolphe, M. Boualam, H. Oumarou Hama, E. Terrer

**Affiliations:** 1 Aix-Marseille Université, MEPHI, Marseille, France; 2 IHU Méditerranée Infection, Marseille, France; 3 Assistance Publique des Hôpitaux de Marseille, APHM, Marseille, France; Connecticut Agricultural Experiment Station, New Haven, Connecticut, USA; Universite Grenoble Alpes, Auvergne-Rhône-Alpes, France; IHU Mediterranee Infection, Marseille, France

**Keywords:** LAMP, environment, *Vibrio* spp., cholera, *Vibrio cholerae*, alkaline lysis, detection

## Abstract

*Vibrio cholerae* (*V. cholerae*), causing deadly cholera, which is responsible for significant public health problems in endemic regions, should be monitored in contaminated environments. Here, we report an integrated protocol incorporating alkaline lysis DNA extraction and loop-mediated isothermal amplification (LAMP) targeting the *V. cholerae rpo*B gene and producing a colorimetric reading within 1 h. This protocol yielded 100% specificity for *V. cholerae* after testing three non-cholerae *Vibrio* species and four *Enterobacteriaceae* as negative controls. A positive signal was detected across a range of 3 × 10^8^ to 3 × 10^3^ cells/mL for four *V. cholerae* isolates within 30–60 min. Following the inoculation of 3 × 10^4^ *V. cholerae* cells into five simulated environmental matrices (soil, sand, seawater, tap water, and anthropogenic effluents), positive results were similarly obtained between 3 × 10^4^ and 3 × 10^3^ cells/mL over the same time interval. In contrast, at the same conditions, *Vibrio alginolyticus* (*V. alginolyticus*) and *Escherichia coli* mock-infected matrices, which were used as negative controls, remained negative. These data suggested that this LAMP protocol could be implemented in the field to monitor *V. cholerae* in environmental matrices at the point of interest in countries affected by this deadly pathogen.

**IMPORTANCE:**

Cholera is an intestinal infection caused by the bacterium *Vibrio cholerae*, resulting in vomiting and acute watery diarrhea. If left untreated, it can cause severe dehydration and death. Although various detection tests have been developed for different types of samples, cholera remains endemic in several countries, particularly where access to safe drinking water is limited and sanitation infrastructure remains inadequate. The objective of our research is to explore different environmental matrices to design a screening system for *V. cholerae* in environmental samples, thus contributing to the development of a system that can be used in the environment.

## INTRODUCTION

*Vibrio* spp. form a group of gram-negative bacteria commonly encountered in aquatic and marine environments. Twelve of them have been reported to be responsible for enteritis after consumption of contaminated water and seafoods (1). One of these species, *Vibrio cholerae* (*V. cholerae*), encodes a viral-sourced toxin cholera (CTXΦ), which alters intestinal NaCl and water homeostasis and is responsible for an acute, deadly dehydration known as cholera (2, 3). *Vibrio parahaemolyticus* (*V. parahaemolyticus*), *Vibrio alginolyticus* (*V. alginolyticus*), and *Vibrio vulnificus* (*V. vulnificus*) additionally cause potentially deadly wound infections, as illustrated by cases of *V. vulnificus* documented during the 1861–1865 civil war in the United States (4). Of the 200 referenced *V. cholerae* serotypes, serotypes O1 and O139 have been largely associated with cholera epidemics and pandemics worldwide (5). Six historical pandemics were documented during the 19th and 20th centuries, caused by two serotypes, O1 and O139, while the current pandemic is due to the El Tor biotype (6). Globalization and transcontinental exchanges promoted the geographical spread of later cholera pandemics, as illustrated by the importation of *V. cholerae* in Haiti following the arrival of blue helmet soldiers from a cholera-endemic area in Nepal, 10 months after the earthquake of 12 January 2010 (7, 8). In Haiti, more than 500,000 cases and 7,025 deaths were reported by the Ministry of Public Health and Population after cholera spread across the country between October 2010 and January 2012, facilitated by inadequate sanitary conditions (9). Between January 2023 and March 2024, a cumulative total of 824,479 cholera cases and 5,900 deaths were reported globally in five World Health Organization (WHO) regions (Eastern Mediterranean Region, African Region, Region of the Americas, South-East Asia Region, and Western Pacific Region) (https://reliefweb.int/report/malawi/multi-country-outbreak-cholera-external-situation-report-13-published-17-april-2024). In Haiti, since October 2022, 13,672 suspected cases of cholera, leading to 283 deaths, have been reported, and between January 2023 and 31 March 2024, 59,027 cases and 792 deaths from cholera were reported by the Ministry of Public Health and Population (MSPP) in all 10 departments (https://www.who.int/emergencies/disease-outbreak-news/item/2022-DON427). In October 2024, a total of 37,363 new cases of cholera and acute diarrhea were reported in 19 countries across three WHO regions (African Region, Eastern Mediterranean Region, and South-East Asian Region) (https://www.who.int/docs/default-source/coronaviruse/situation-reports/20241111_multi-country_outbreak-of-cholera_sitrep_-20).

Affected populations are the main reservoirs for contagious *V. cholerae*, which can also be detected in some environmental matrices, mostly water-related environments (10, 11). *V. cholerae* has been detected in freshwater rivers and lakes, as well as in estuarine and marine environments, in which *Vibrio* spp. cells form halotolerant biofilms on abiotic and biotic surfaces such as zooplankton and phytoplankton (12) The detection of environmental *V. cholerae* is currently based on in-lab techniques using isolation culture and immunological tests (13). Environmental *V. cholerae* has also been detected using immunofluorescence (14), PCR (15), and loop-mediated isothermal amplification (LAMP) assays, for which several protocols have been developed targeting *V. cholerae*-specific *ctx*A, *rfb*N, and *omp*W genes (16). All these techniques require laboratory facilities and machines, which are usually lacking in remote areas and in the regions most affected by cholera.

In this study, we develop a LAMP-*V. cholerae* amplification system specifically targeting the *V. cholerae rpo*B gene after rapid alkaline lysis extraction. This protocol aimed to overcome limitations of the colorimetric detection of LAMP-amplified *V. cholerae* after simple alkaline lysis of the pathogen in its matrix, with a view to its point-of-interest implementation to monitor environmental *V. cholerae* in endemic countries.

## RESULTS

### DNA extractions

Extracted DNA was amplified by SYBR PCR, showing Ct values proportional to the cascade dilutions of DNA. Ct values greater than 35 were considered negative because the results obtained showed no proportionality between Ct values equal to or greater than 34. This result shows that the alkaline lysis extraction method is effective on our samples and shows very precise DNA extraction (Table 1).

**TABLE 1:** CT value showing DNA extraction by alkaline lysis after RT-PCR SYBR

Dilutions	10^8^	10^7^	10^6^	10^5^	10^4^	10^3^	10^2^
CT value	15.34	18.35	21.52	24.57	27.49	31.03	34.05

### *V. cholerae* isothermal amplification sensitivity and specificity

**Fig 1:**
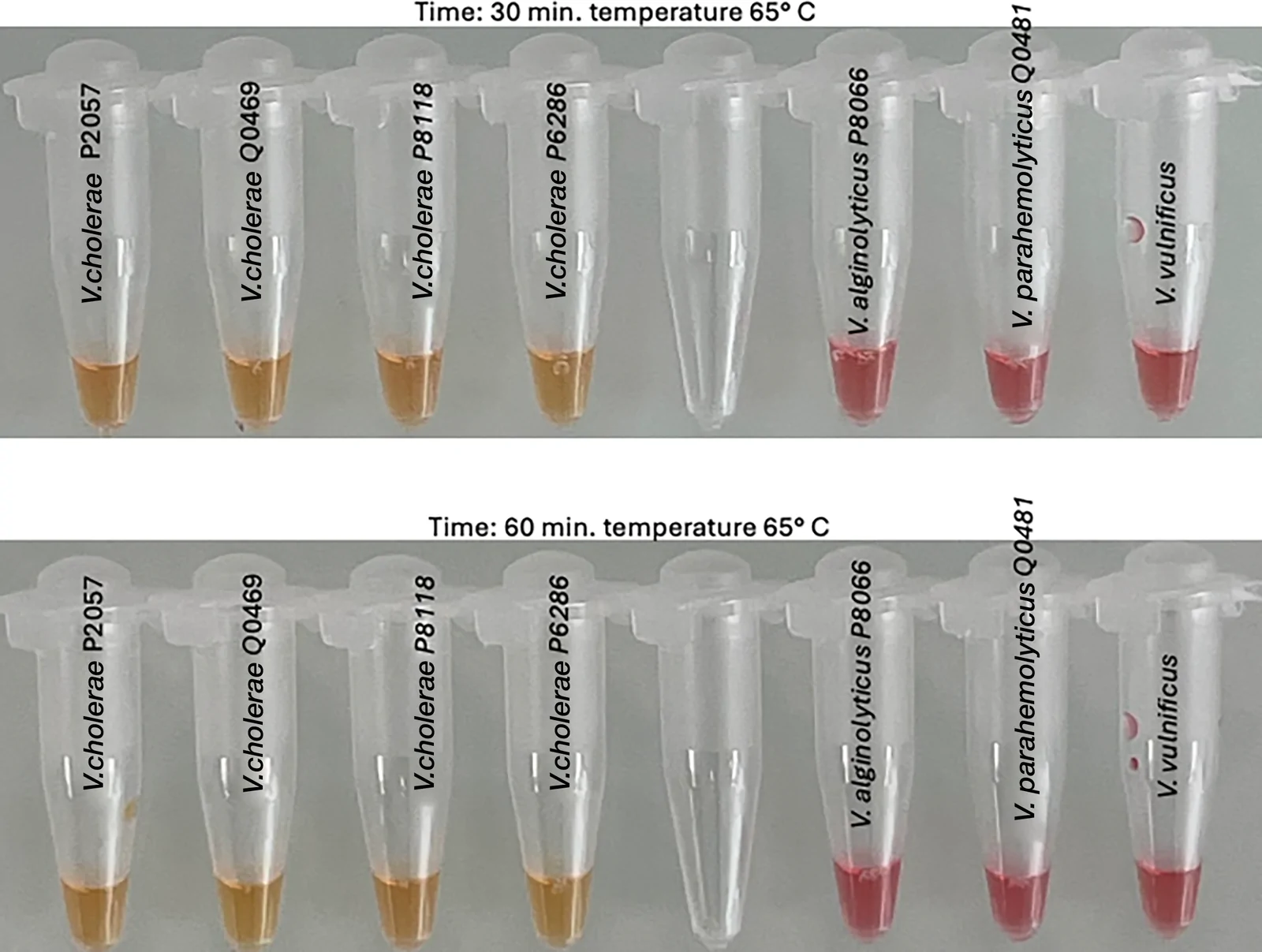
LAMP optimization of specificity LAMP-*V. cholerae* system for each *V. cholerae* strain, with negative controls: three other non-*V*. *cholerae* (*V. alginolyticus, V. parahaemolyticus, V. vulnificus*). This result shows LAMP-*V. cholerae* is specific and designated primers only target *V. cholerae*.

**Fig 2:**
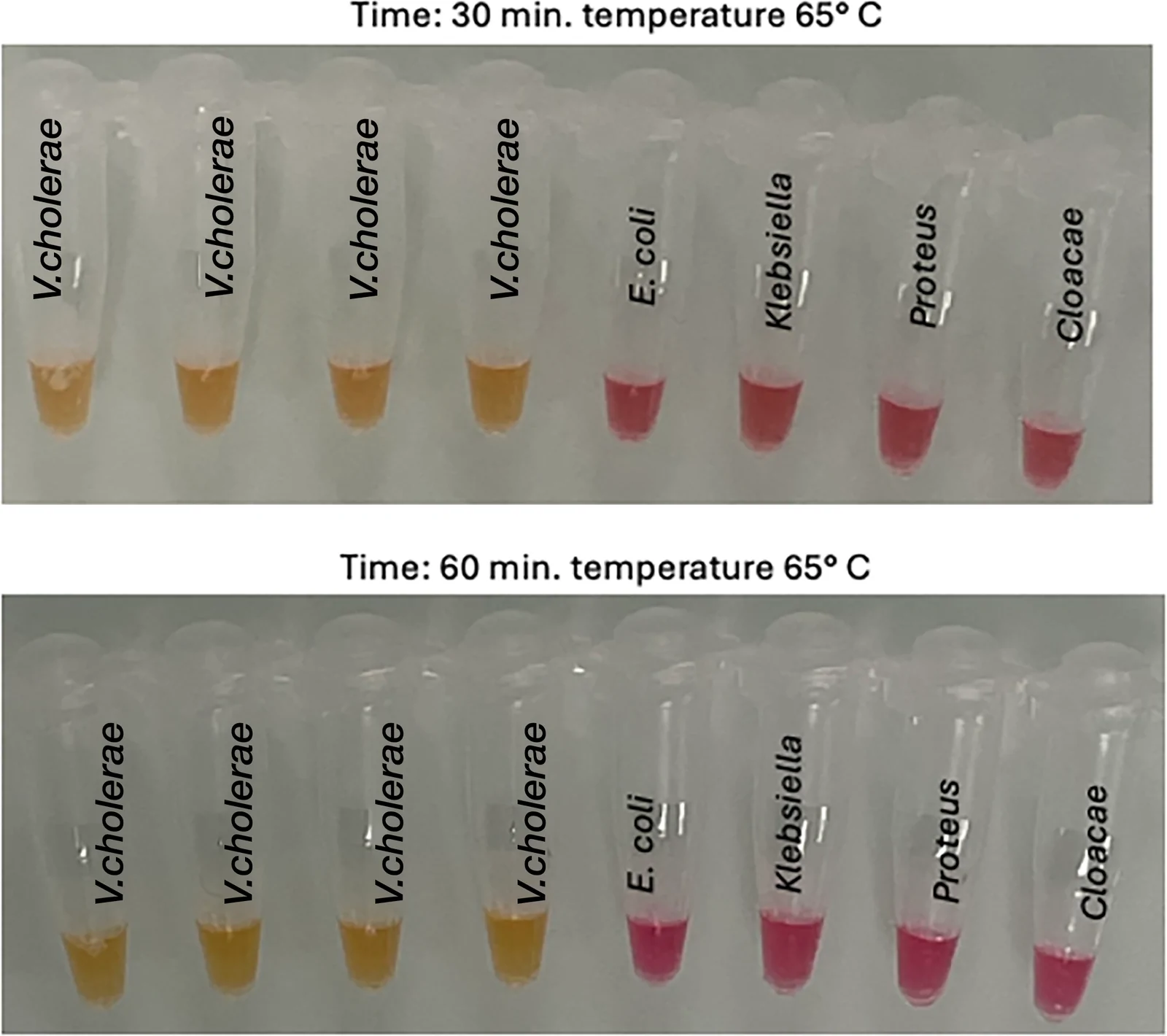
LAMP optimization on *Vibrio cholerae*, bar containing the mix 50 µL with DNA *klebsiella, P. mirabilis, E. cloacae*, the *E. coli* strain, and one extraction control content as negative. This result shows LAMP-*V. cholerae* is specific and designated primers only target *V. cholerae*.

**Fig 3:**
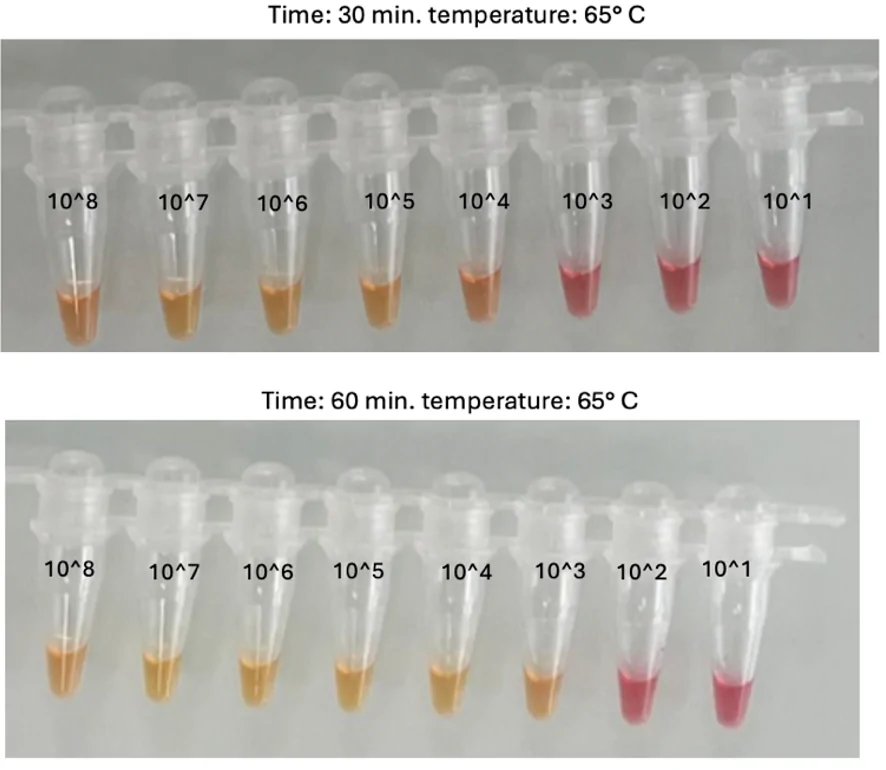
Sensibility of the LAMP-*V. cholerae* system on cascade dilution shows sensitivity up to 10^3^ cells/mL and a sensitivity of 1,000 cells/mL at dilution. The resulting LAMP-*V. cholerae* is sensitive to very small amounts of DNA.

After a 30-min reaction, *V. cholerae* DNA displayed a change in color from pink to yellow, indicating a positive amplification reaction. After a 60-min reaction, the yellow color intensified (Fig. 1). This observation held true for the four tested *V. cholerae* strains, whereas *V. alginolyticus*, *V. parahaemolyticus,* and *V. vulnificus* consistently yielded negative results. These data confirmed the specificity of the LAMP assay for *V. cholerae* among the *Vibrio* genus. Furthermore, *Escherichia coli*, *E. cloacae*, *K. aerogenes*, and *P. mirabilis* used as negative controls, consistently yielded a pink color after a 60-min reaction, indicating no amplification (Fig. 2). Under these same conditions, a positive detection occurred up to 10^3^ dilutions sensitivity of the LAMP-*V. cholerae* bacteria (Fig. 3).

### *V. cholerae* detection in mock-contaminated environmental matrices

The color of tap water, seawater, effluent, beach sand, and local soil spiked with 10^4^ cells/mL of *V. cholerae* changed from initially pink to yellow after 30 min of reaction. Each tube remained yellow for up to 60 min at 65°C, indicating a positive amplification. For all control matrices spiked with 10^4^ CFU/mL of *V. alginolyticus*, *E. coli* retained its pink color even after 60 min of reaction, indicating a negative amplification (Fig. S1 to S5). Each reaction was repeated by three different experimenters to confirm the specificity of the LAMP-*V. cholerae* system.

### RT-PCR SYBR detection in mock-contaminated environmental matrices

Amplification of DNA extraction from tap water, seawater, effluent, beach sand, and local soil spiked with 10^4^ cells/mL of *V. cholerae*, to compare RT-PCR to LAMP-*V. cholerae*. Ct values showed proportionality to the serial dilutions of DNA. Ct values greater than 35 were considered negative because the results obtained showed no proportionality between Ct values equal to or greater than 34 (Table 2).

**TABLE 2:** LAMP-*V. cholerae* shows a constant detection limit (10^4^ copies) for all samples, confirming its specificity under various environmental conditions; RT-PCR has high Ct (>34), which indicates a low amount of target DNA and possible inhibition due to the template; tap water and effluents offer the best performance (few inhibitors); soil, sand, and seawater show inhibition effects related to the chemical and organic complexity of the samples^*a*^

Comparison table LAMP-*V. cholerae* and RT-PCR-*V. cholerae*					Comparative methods	
Sample	10^4^	10^3^	10^2^	10^1^	LAMP	RT-PCR
Tap water	LAMP(+) and RT-PCR (+)	LAMP(−) and RT-PCR (+)	LAMP(−) and RT-PCR (+)	LAMP(−) and RT-PCR (+)	Detection limit 10^4^	Ct = 32.93 to 101
Effluents	LAMP(+) and RT-PCR (+)	LAMP(−) and RT-PCR (+)	LAMP(−) and RT-PCR (+)	LAMP(−) and RT-PCR (+)	Detection limit 10^4^	Ct = 34.28 to 101
Soil	LAMP(+) and RT-PCR (−)	LAMP(−) and RT-PCR (−)	LAMP(−) and RT-PCR (−)	LAMP(−) and RT-PCR (−)	Detection limit 10^4^	Ct = 36.09 to 104
Sea water	LAMP(+) and RT-PCR (−)	LAMP(−) and RT-PCR (−)	LAMP(−) and RT-PCR (−)	LAMP(−) and RT-PCR (−)	Detection limit 10^4^	Ct = 36.43 to 104
Sand	LAMP(+) and RT-PCR (+)	LAMP(−) and RT-PCR (+)	LAMP(−) and RT-PCR (−)	LAMP(−) and RT-PCR (−)	Detection limit 10^4^	Ct = 36.43 to 10^2^

^*a*^ The LAMP method is more tolerant and fast, but RT-PCR remains more accurate for fine quantification.

## DISCUSSION

Combining rapid alkaline lysis and the LAMP colorimetric test demonstrated a high capacity for the specific detection of *V. cholerae* across five different organic matrices representative of natural ecosystems. Before DNA extraction, samples were not decontaminated or sterilized prior to inoculation, to preserve microbial diversity and inhibitor loading typical of environmental matrices. This methodological choice aimed to test the robustness and specificity of the LAMP system in more representative field conditions, rather than in an artificially purified environment. The alkaline lysis protocol proved to be an effective extraction method for *V. cholerae* strains, various contaminated environmental matrices, and negative control samples. The speed of the system developed here relies on low-cost materials and basic technical skills. Consequently, this procedure could be deployed in cholera-endemic countries and regions to rapidly trace the environmental presence of *V. cholerae* back to infected individuals. This would enable both a swift medical response for affected individuals, when necessary, and a public health intervention aimed at limiting the spread of the infection. However, these observations obviously do not guarantee that un-mastered field conditions including parameters such as physical and chemical characteristics of environmental samples such as temperature, acidity, potential inhibitors would not influence the limit of detection. Some limitations must be recognized; the trials were carried out under controlled experimental conditions and on a limited number of *V*. *cholerae* strains and non-target species, which does not yet allow a full evaluation of the specificity of the test in the face of environmental bacterial diversity. The presence of natural inhibitors (organic matter, salts, clays, etc.) in the actual samples could affect the effective sensitivity of the test, despite the performance observed in the laboratory.

The LAMP method, although extremely sensitive, does not distinguish between viable and non-viable bacterial DNA, which can lead to an overestimation of environmental risk. To obtain results that are close to the field, the environmental samples were used in their raw state without treatment or decontamination. Validation in real field conditions, on a wider panel of samples and isolates from endemic areas, will be required to confirm the robustness, specificity, and transferability of the system.

The current PCR-based detection approach is time-consuming and primarily requires an expensive thermocycler for amplification, with a total test duration of at least three hours (17). Developing a rapid, sensitive, and cost-effective method for detecting environmental pathogens is essential to improve monitoring and prevention. Unlike PCR, the isothermal LAMP technique can be performed at a constant temperature of 65°C within 30–60 min, without the need for fluorescent probes or costly equipment. Moreover, it preserves DNA integrity by preventing its denaturation (18).

The alkaline lysis extraction protocol was chosen for its simplicity and speed, allowing DNA extraction in the field in less than 30 min. The sensitivity of our system was evaluated on simulated contaminated environmental matrices, demonstrating a strong detection capability. DNA extraction yielded positive results on tap water, seawater, effluents, beach sand, and local soil. These findings confirm that *V. cholerae* can be detected in various environmental samples using the LAMP system described in this study.

This study presents the development of a LAMP system for the rapid detection of *V. cholerae* in different environmental matrices (seawater, sand, soil, tap water, and anthropogenic effluents). The results demonstrate the feasibility and analytical specificity of the method, with a detection limit adapted to environmental monitoring applications, and it is precisely for this reason that, in the present study, the samples were not treated or decontaminated, to assess the intrinsic specificity of the system under conditions as close as possible to environmental reality. While promising, the method would still require field validation, under real-world environmental conditions, to confirm operational performance and reliability at scale. It also highlights the applicability of this approach in field settings, emphasizing its practicality, since it only requires an isothermal heat source at 65°C. Additionally, results can be observed with the naked eye through a color change, simplifying interpretation, and the cost per test could be estimated at £2.66 (19). Table 3 summarizes the various detection methods for *V. cholerae*, and it clearly shows that the LAMP assay is the most suitable for field deployment, given its minimal equipment requirements and ease of use for rapid screening.

**TABLE 3:** Overview of *Vibrio cholerae* detection methods, detailing their analytical principles, sample types, indicative lead times, result quality, key strengths, and major limitations

Method	Principle	Samples	Incative lead times	Quality	Key strengths	Limitations	References
Culture	APW enrichment and selective isolation on TCBS	Stool, Them, Food, Surface Water	24–72 h	Moderate sensitivity; High specificity	“Gold standard” for possible confirmation, antibiogram and genotyping	Lack of sensitivity under low load or VBNC status	(20)
PCR	Amplificaation ADN	Water, sediment, complex matrices	4–8 h	Sensitivity/Specificity Good (depends on primers)	Simple, inexpensive, multiplex possible	Not very quantitative; Sensitive to inhibitors	(21)
qPCR(real-time)	Quantitative detection	Water (tap/sea/waste)	2–3 h	High sensitivity and specificity; quantifiable	Speed, quantification, multiplex	dead cell DNA; matrix inhibition	(22)
LAMP	Isothermal amplification (60–67°C) targets specific gene	Water, sediment	30–60 min	High sensitivity; fast	Very suitable for the terrain; Inhibitor tolerance; visual readout/fluorescence	Contamination; qualitative non-quantitative; False positive	(16)
Immunological test	Immunochromatography	Stool (clinical)	15–30 min	Sensitivity/Specificity varies according to batch and context	Very fast field screening	Variable return: Culture/qPCR confirmation required	(23)
ELISA (toxin/antigen)	Antigen detection	Stool	2–5 h	Correct sensitivity; specific to the targeted antigen	Standardized, multi- well	Antigen specificity; less useful under very low load	(21)

### Conclusion

This study is a proof of concept demonstrating the analytical feasibility and specificity of the LAMP system for the rapid detection of *V. cholerae* in different environmental matrices. The results obtained confirm a potential approach for future applications in environmental monitoring and early detection of cholera. Further testing with real environmental samples from cholera-endemic regions will provide an excellent opportunity to evaluate and implement our method in real-world conditions, while adhering to ethical guidelines and informed consent requirements.

## MATERIALS AND METHODS

### *Vibrio* spp. strains

*V. cholerae* CSURP0469, *V. cholerae* CSURP2057, *V. cholerae* CSURP6286, *V. cholerae* CSURP8118, *V. vulnificus* CSURQ6273, *V. alginolyticus* CSURP4248, and *V. parahaemolyticus* CSURP8066 retrieved from the Collection de Souches de l’Unité des Rickettsies “CSUR’’ (IHU Méditerranée Infection, Marseille, France), were handled in an NSB-2 laboratory in keeping with regulations in France at the time this study was conducted (24). These strains were cultured on thiosulfate-citrate-bile salts-sucrose agar (TCBS) (bioMérieux, Marcy l'Etoile, France), incubated for 24 h at 37°C under a 5% CO_2_ enriched atmosphere. They were then further sub-cultured on 5% sheep’s blood Columbia agar (COS, bioMérieux) for an additional 24 h in the same conditions. Bacterial suspensions were prepared in NaCl 0.85% (bioMérieux), calibrated at 1 McFarland (equivalent to 3 × 10^8^ cells/mL), and a series of 10-fold dilutions from 10^1^ to 10^6^ prepared in NaCl 0.85% were stored at −80°C. Accurate identification of each isolate was confirmed by matrix-assisted laser desorption ionization time-of-flight mass spectrometry (MALDI-TOF-MS; Microflex LT, Bruker Daltonik), as previously described (25).

### DNA extraction

DNA extraction was performed following one protocol: alkaline lysis protocol, 100 μL of bacterial suspension at 1 McFarland were mixed with 100 μL of NaOH at 2 g/L (pH = 12.45) (Fresenius Kabi AG, Bad Homburg, Germany) and incubated at room temperature for 3 min, then 100 μL of Tris-HCl solution at 1 g/L (pH = 5.4) (Fresenius Kabi AG, Geel, Belgium) was added to neutralize the mixture. The resulting final volume of 300 μL was partially used for subsequent procedures, while the remaining aliquots were stored at –20°C until use. For amplification, serial dilutions were made up to 10^8^–10^1^. Extracted DNA was quantified by Qubit assay before the LAMP-*Vibrio* test and RT-PCR SYBR. To further ensure that the alkaline lysis protocol killed the *Vibrio* cells, DNA extracted by alkaline lysis was cultured for 24 h on COS agar (COS, bioMérieux) at 37°C, in triplicate with un-extracted bacterial suspensions used as positive control.

### Design of the LAMP primers

Six LAMP primers: FIP-BIP inner primers, F3-B3 outer primers and LF-LB loop primers targeting *V. cholerae rpo*B gene were designed using the NEB LAMP primer Design Tool (https://lamp.neb.com), and their specificity *in silico* was tested using NCBI Blastn (https://blast.ncbi.nlm.nih.gov/Blast.cgi) has been tested (Table 4); and primers show a hook only with *V. cholerae* (Fig. S6).

**TABLE 4:** LAMP primers used in this study

LAMP primers used in this study: rpoB	
LF (rpoB)	CAGTGCAGAGATGCGATCTGTC
LB (rpoB)	AGTCTATTCTTCTCTGCAGAGCGTT
F3 (rpoB)	ACGGTCCGTTCATGTCAGA
B3 (rpoB)	CGACCGATTGAGCTGTTGAA
FIP (rpoB)	GGCGCATCATGCGGTAGATCTCCCCTGCGTGTTGATAGCAC
BIP (rpoB)	GCCGCGGAATCGCTGTTTGATCATACGGCCAACAGTCGA
LF (rpoB)	CAGTGCAGAGATGCGATCTGTC

### DNA extraction validation by real-time PCR amplification

For amplification F3-B3 outer primers loop primers targeting *V. cholerae rpoB* gene, extracted DNA was incorporated into a 20 μL amplification mix containing 10 μL of Light Cycler 480 SYBR Green Master (Roche Diagnostic GmbH Manhen, Germany), 3.5 μL of water, 0.5 μL each of the F3 and B3 LAMP primers complemented with 5 μL of DNA extraction. A DNA-free mixture was used as a negative control. Amplification consisting of 10-min polymerase activation at 95°C followed by 39 cycles of 10-s denaturation at 95°C and 30-s hybridization at 60°C, was performed on a BIO-RAD T100 Thermal Cycler (Bio-Rad Inc., Hercules, CA, USA).

### *V. cholerae* isothermal amplification, sensibility, and specificity tests

The loop-mediated isothermal amplification *V. cholerae* assay was performed: primers were prepared from a 1,000 μM stock solution. Each 50 μL reaction contained 16 μmol/L of inner primers (FIB and BIP), 2 μmol/L of outer primers (F3 and B3), 4 μmol/L of loop primers (LF and LB), 44 μL of water (Invitrogen, Paisley, UK) resuspended in the RT-LAMP colorimetric master mix (Biotechnology Co, Wuhan, Hubei, China) and 1 μL of DNA. Isothermal amplifications consisted of a single 60-min step at 65°C performed in a T100 Thermal Cycler (Bio-Rad Inc.). One McFarland *V. cholerae* suspension was diluted from 10^8^ to 10^1^ in the extraction solution (NaOH and Tris-HCl) to measure the sensitivity of the LAMP system. Each LAMP reaction was read twice at 30 and 60 min. Negative controls consisted in *Escherichia coli, Enterobacter cloacae, Klebsiella aerogenes*, *Proteus mirabilis*, one extraction control, and three non-*V*. *cholerae* (*V. alginolyticus*, *V. parahaemolyticus,* and *V. vulnificus*) strains, the DNA of which had been extracted following the same extraction and reaction procedures. In parallel, four *V. cholerae* strains were used as positive controls to test the specificity of LAMP-*V. cholerae*.

### *V. cholerae* detection in mock-contaminated environmental matrix

A total of five environmental matrices in Marseille were used: soil, sand, seawater, tap water, and anthropogenic effluents. None of these samples were sterilized or decontaminated prior to seeding (*spiking*) with *V. cholerae*, to evaluate the performance of the system under near-field environmental conditions. For the environmental matrices, we used the following volumes: 1.5 mL of tap water, 1.5 mL of sea water, 1.5 mL of effluents, 0.5 g of beach sand, and 0.5 g of local soil. The soil and beach sand were mixed with 1 mL of DNAse/RNAse-free water to form a slurry, then 100 μL of bacterial suspension was added, followed by centrifugation at 12,000 rpm (13.8 g) for 1 min, and the supernatant was collected. After turbidity adjustment using the McFarland standard, the corresponding concentration was verified by culture-based enumeration. Bacterial suspensions adjusted to 1 McFarland unit (≈3 × 10^8^ cells/mL) were prepared and subjected to successive 10-fold dilutions (10⁷–10¹). Each dilution was plated on Columbia blood agar (COS) and incubated at 37°C for 24 h to determine colony-forming units (CFU), thereby confirming the agreement between the McFarland estimation and the actual bacterial concentration (Fig. S7). For each matrix, 100 μL was spiked with 100 μL of a 10⁴ CFU/mL *V. cholerae* suspension and, in parallel, with 100 μL of a 10^4^ CFU/mL non-cholera *Vibrio* suspension. From each inoculated matrix, 100 μL was collected for DNA extraction using the alkaline lysis protocol. One microliter of the resulting 300 μL DNA extract was incorporated into the LAMP-*V. cholerae* assay and RT-PCR. Each reaction was performed in triplicate, by different operators who received the same protocol, the same reactants, and the same equipment. The independent replicates all yielded similar results, confirming the reproducibility and robustness of the LAMP system used.

## Supplementary Material

Reviewer comments

## EDITOR'S NOTE

**Ed. Note**: A potential conflict of interest was identified after acceptance of this paper, and the senior editor conducted an additional review to ensure the integrity and fairness of the peer review process.

## SUPPLEMENTAL MATERIAL

Figures S1 to S7 spectrum.02753-25-s0001.docx Figure S1: Samples of soil, contaminated with *V. cholerae*, after amplification at 65°C for one 60 minutes on thermocycler. The results show sensitivity for the sample containing *V. cholerae*, coloration yellow. Figure S2: Anthropogenic effluents contaminated with *V. cholerae*, after amplification at 65°C for 60 minutes on a thermocycler. The results show sensitivity for the sample containing *V. cholerae*, coloration yellow. Figure S3: Tap water contaminated with *V. cholerae*, after amplification at 65°C for 60 minutes on a thermocycler. The results show sensitivity for the sample containing *V. cholerae*, coloration yellow. Figure S4: Sea water contaminated with *V. cholerae*, after amplification at 65°C for 60 minutes on a thermocycler. The results show sensitivity for the sample containing *V. cholerae*, coloration yellow. Figure S5: sand contaminated with *V. cholerae*, after amplification at 65°C for 60 minutes on a thermocycler. The results show sensitivity for the sample containing *V. cholerae,* coloration yellow. Figure S6: Screenshot NCBI blastn *in silico* of the primers. Figure S7: Bacterial suspensions of 1 McFarland density (≈ 3 × 10⁸ cells/mL) were prepared, then subjected to successive decimal dilutions. Each dilution was inoculated on Columbia blood agar (COS) and incubated at 37°C for 24 h for enumeration.

OPEN PEER REVIEW reviewer-comments.pdf An accounting of the reviewer comments and feedback.

ASM does not own the copyrights to Supplemental Material that may be linked to, or accessed through, an article. The authors have granted ASM a non-exclusive, world-wide license to publish the Supplemental Material files. Please contact the corresponding author directly for reuse.
