# Supplementary material for: Isothermal amplification and colorimetric detection of Vibrio cholerae in environmental matrices
Source: Microbiol Spectr. 2026 May 21;14(7):e02753-25. doi: 10.1128/spectrum.02753-25 (PMC13340027; doi:10.1128/spectrum.02753-25)
Supplement: Figures S1 to S7 — Figure S1: Samples of soil, contaminated with V. cholerae, after amplification at 65°C for one 60 minutes on thermocycler. The results show sensitivity for the sample containing V. cholerae, coloration yellow. Figure S2: Anthropogenic effluents contaminated with V. cholerae, after amplification at 65°C for 60 minutes on a thermocycler. The results show sensitivity for the sample containing V. cholerae, coloration yellow. Figure S3: Tap water contaminated with V. cholerae, after amplification at 65°C for 60 minutes on a thermocycler. The results show sensitivity for the sample containing V. cholerae, coloration yellow. Figure S4: Sea water contaminated with V. cholerae, after amplification at 65°C for 60 minutes on a thermocycler. The results show sensitivity for the sample containing V. cholerae, coloration yellow. Figure S5: sand contaminated with V. cholerae, after amplification at 65°C for 60 minutes on a thermocycler. The results show sensitivity for the sample containing V. cholerae, coloration yellow. Figure S6: Screenshot NCBI blastn in silico of the primers. Figure S7: Bacterial suspensions of 1 McFarland density (≈ 3 × 10⁸ cells/mL) were prepared, then subjected to successive decimal dilutions. Each dilution was inoculated on Columbia blood agar (COS) and incubated at 37°C for 24 h for enumeration. [file spectrum.02753-25-s0001.docx]

**Supplementary Data**

**Supplemental Figure:**

**
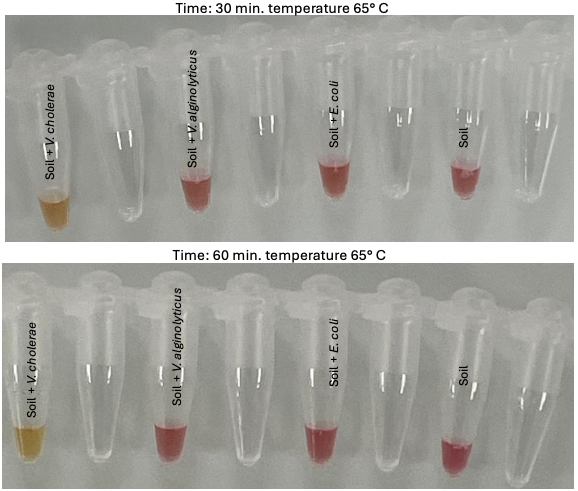
**

**Figure S1:** samples of soil, contaminated with *V. cholerae*, after amplification at 65 °C for one 60 minutes on thermocycler. The results show sensitivity for the sample containing *V. cholerae,* coloration yellow.


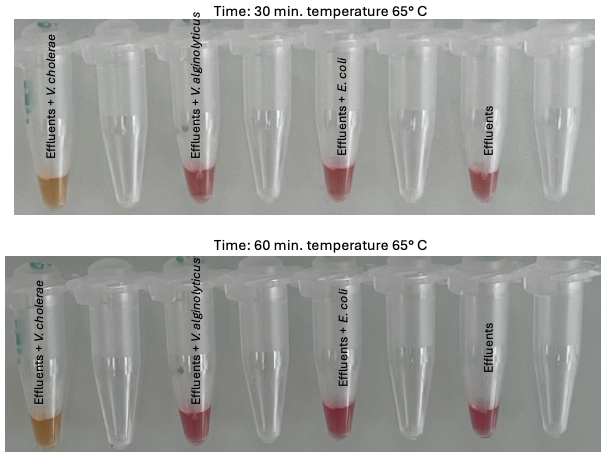


**Figure S2:** anthropogenic effluents contaminated with *V. cholerae*, after amplification at 65 °C for 60 minutes on a thermocycler. The results show sensitivity for the sample containing *V. cholerae,* coloration yellow.


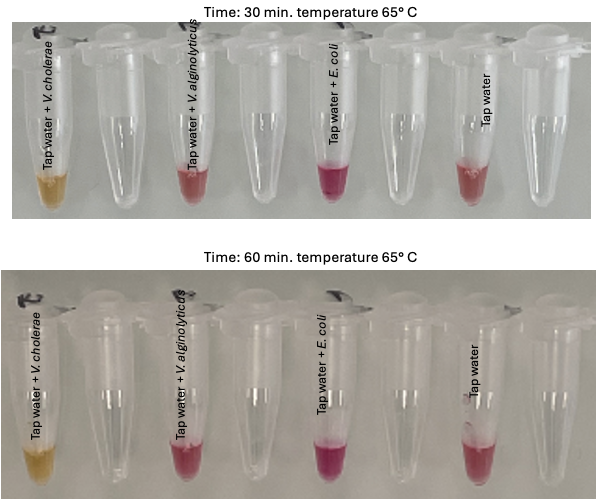


**Figure S3:** tap water contaminated with *V. cholerae*, after amplification at 65 °C for 60 minutes on a thermocycler. The results show sensitivity for the sample containing *V. cholerae,* coloration yellow.

**
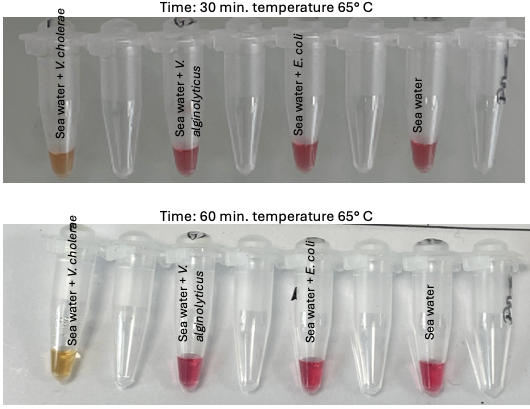
**

**Figure S4:** sea water contaminated with *V. cholerae*, after amplification at 65 °C for 60 minutes on a thermocycler. The results show sensitivity for the sample containing *V. cholerae,* coloration yellow


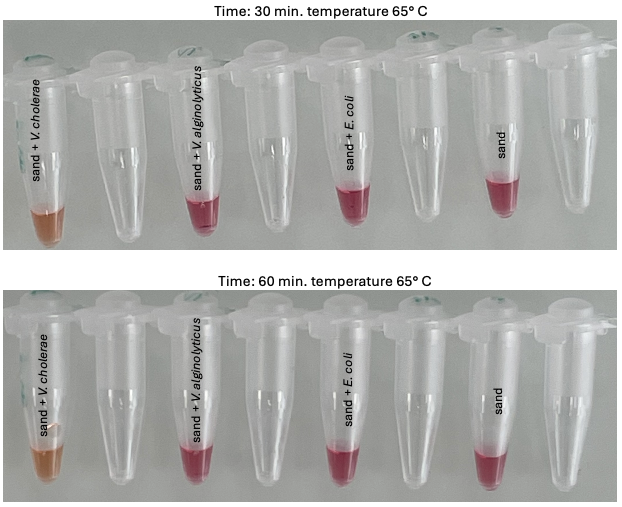


**Figure S5:** sand contaminated with *V. cholerae*, after amplification at 65 °C for 60 minutes on a thermocycler. The results show sensitivity for the sample containing *V. cholerae,* coloration yellow.

**Figure S6:** primer specificity


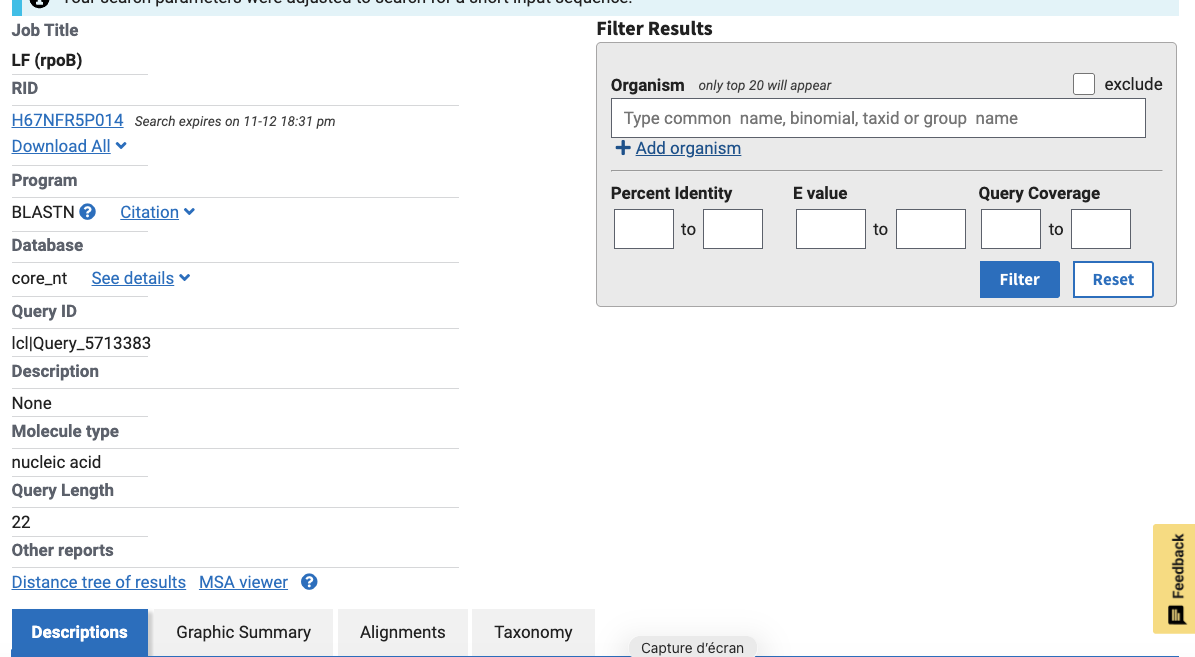


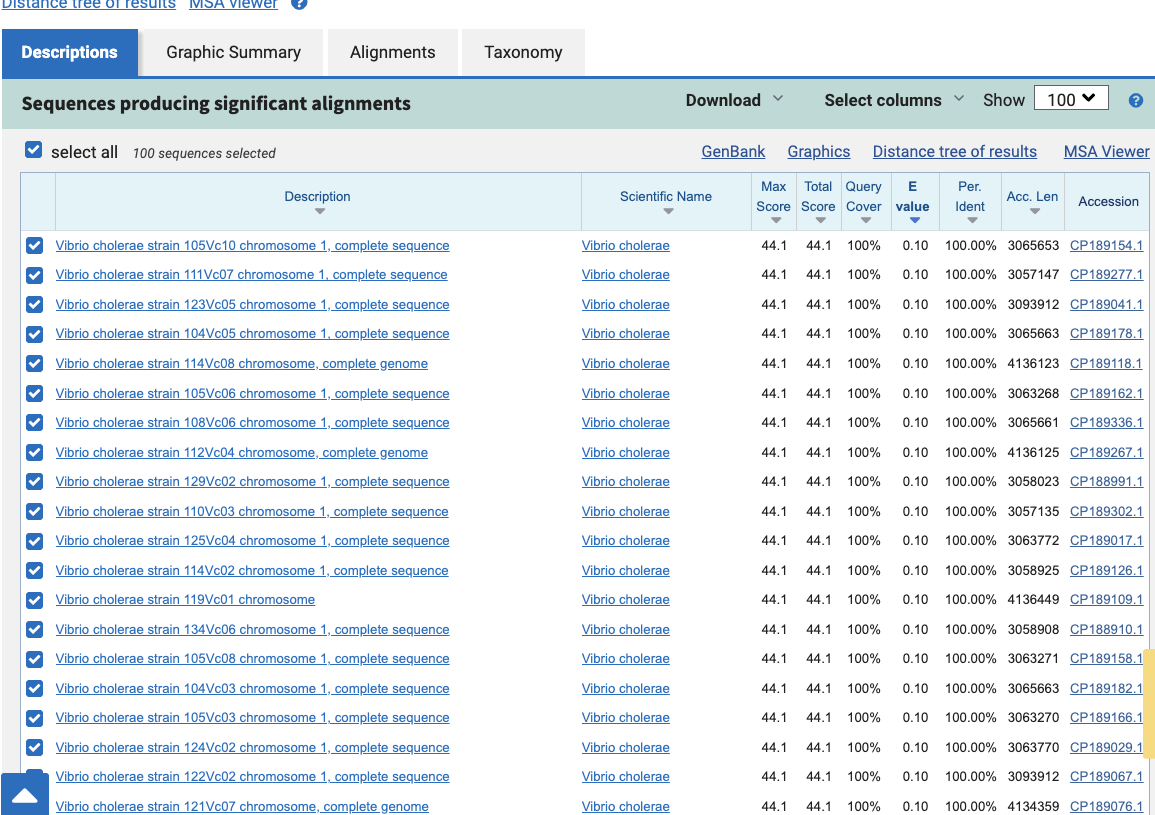


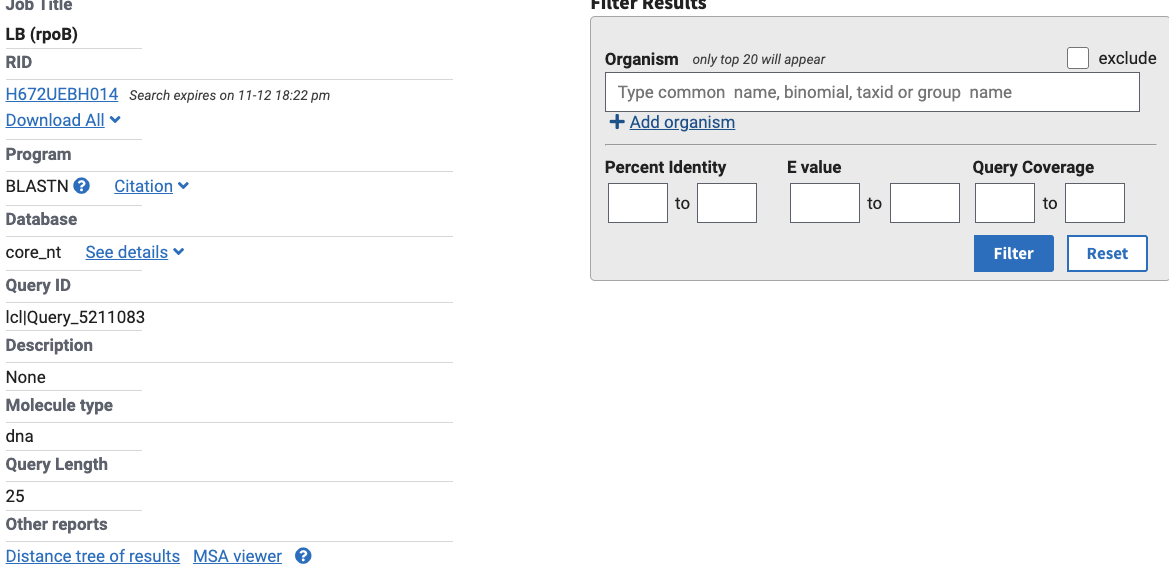


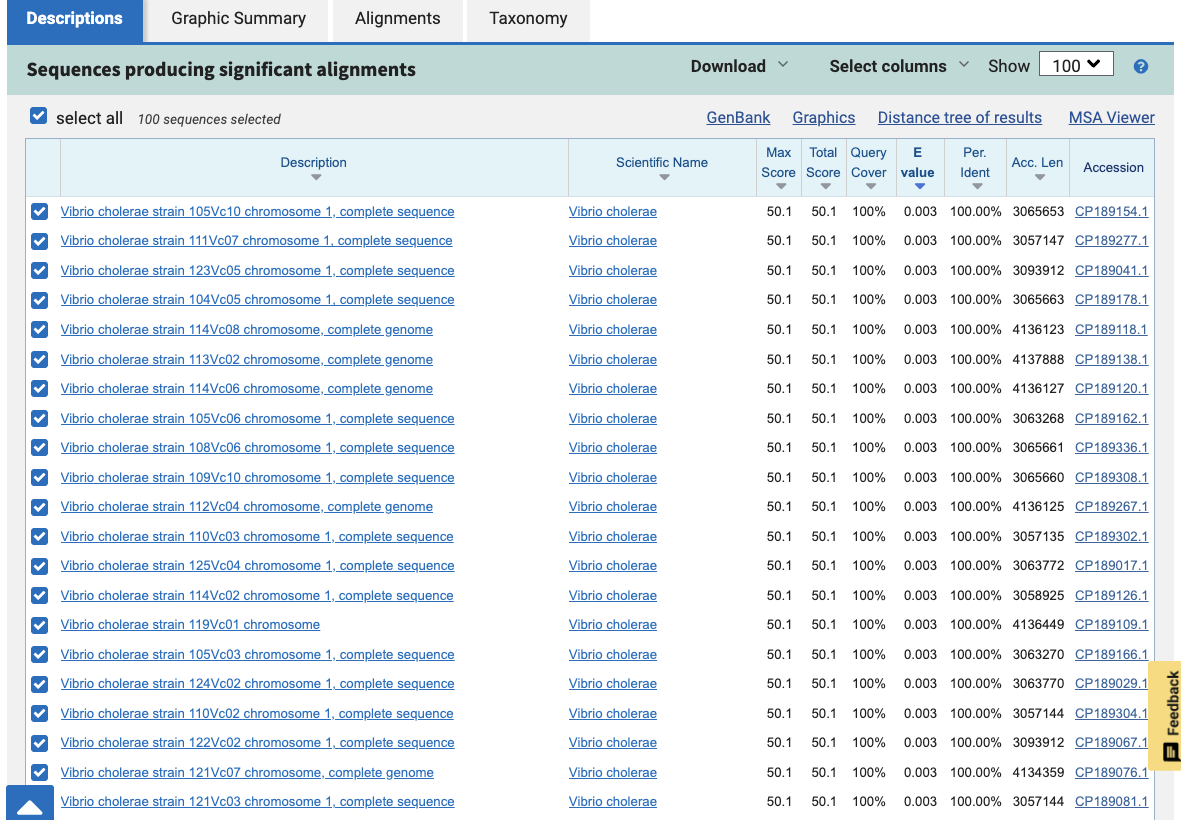


**
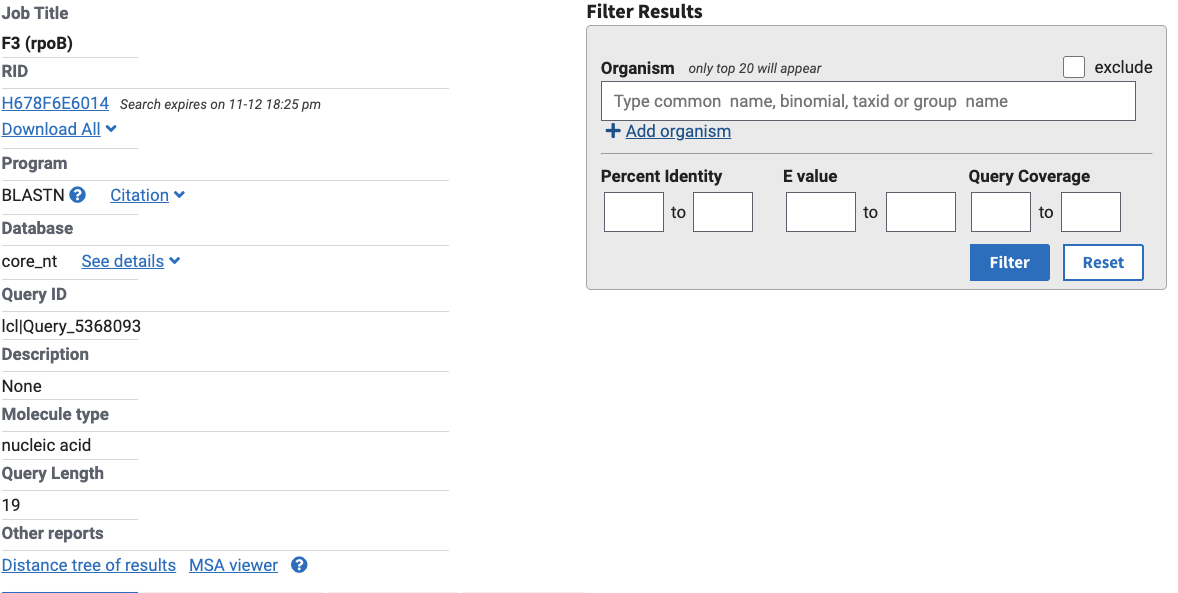
**

**
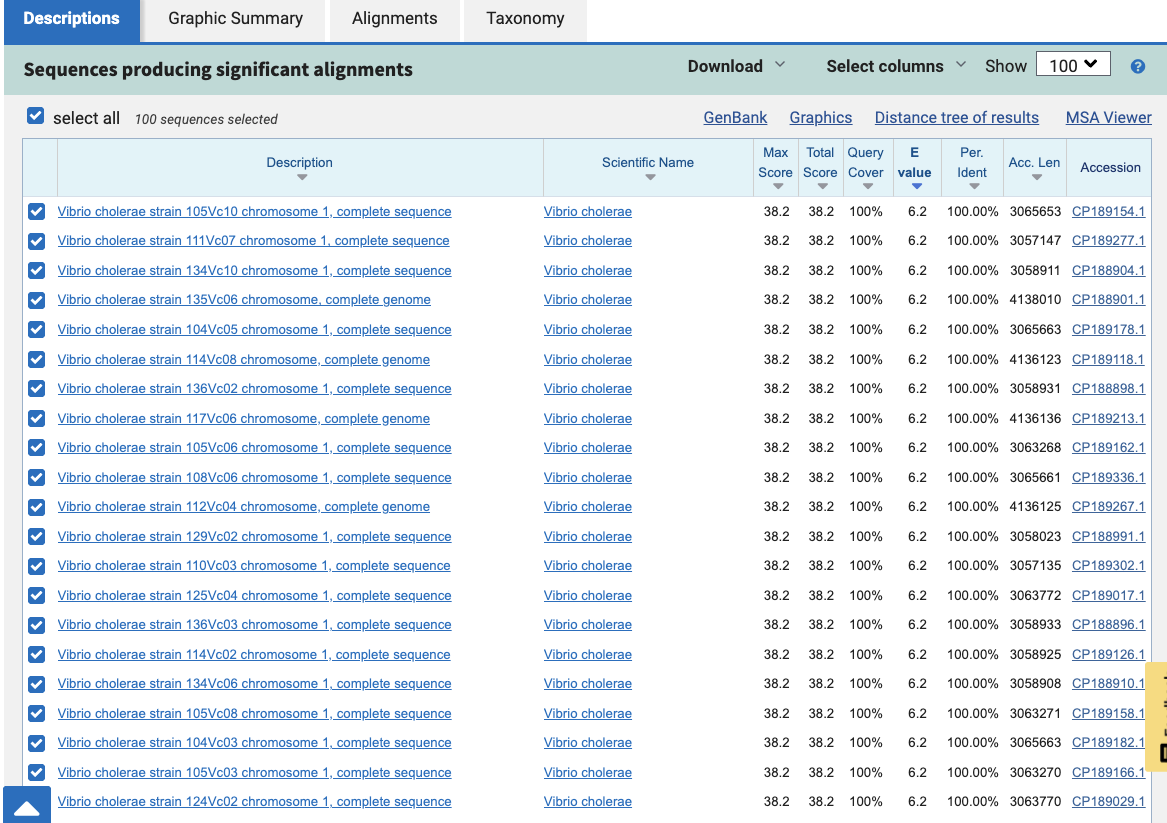
**

**
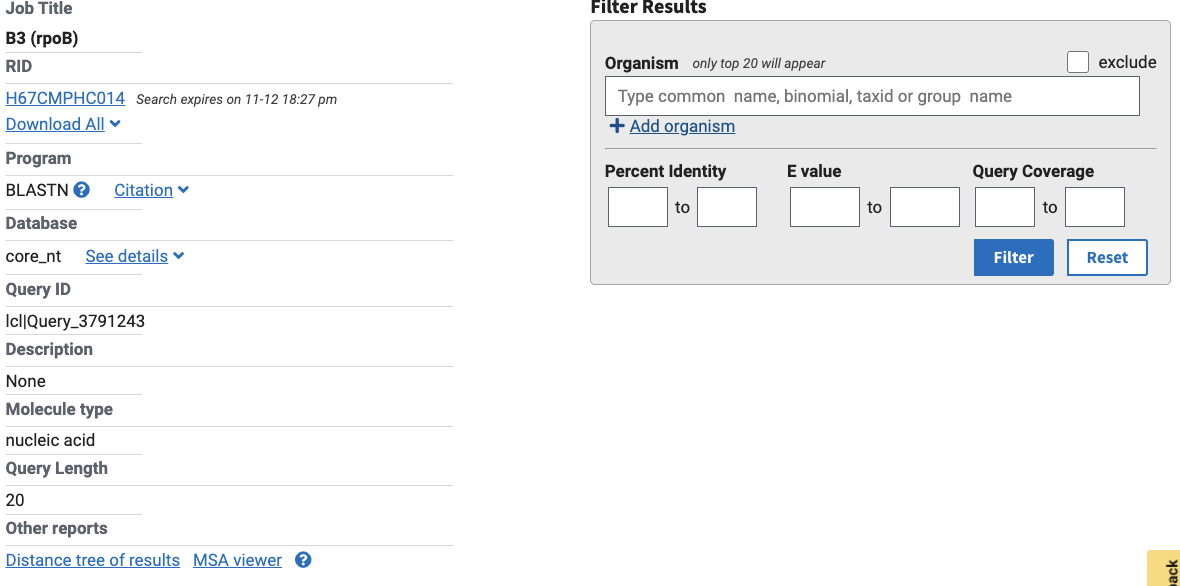
**

**
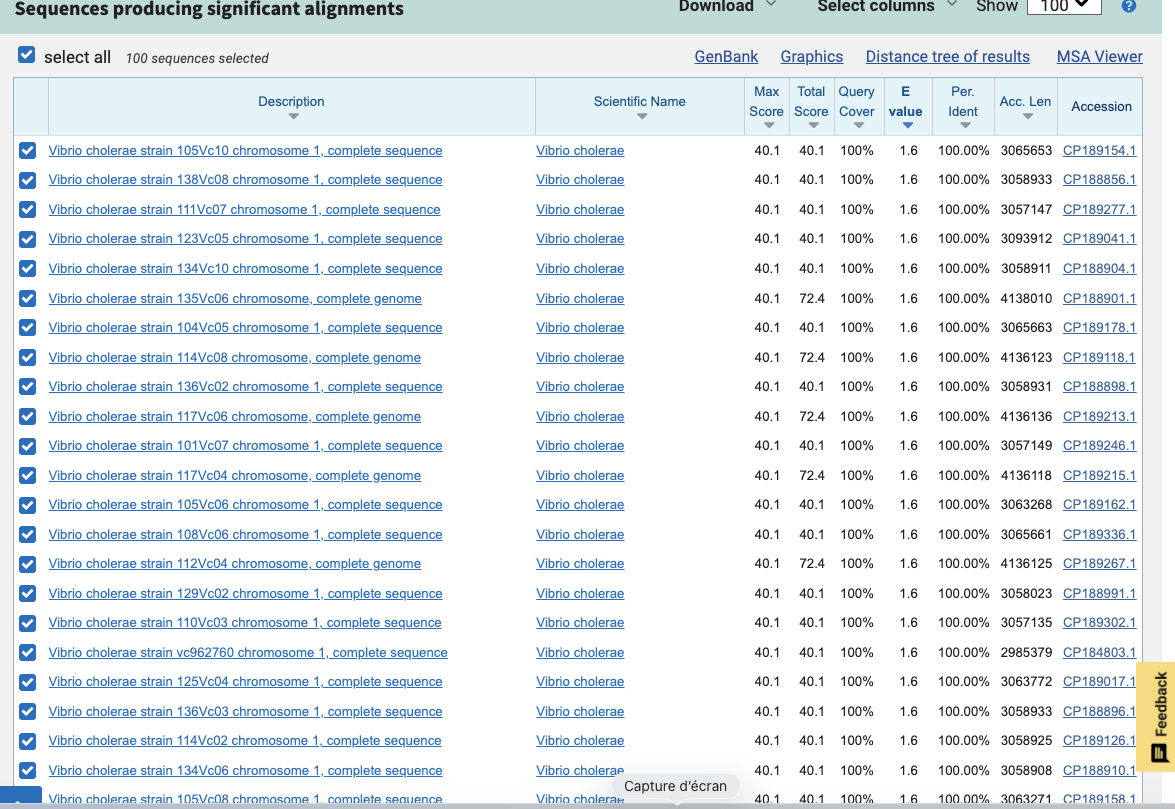
**


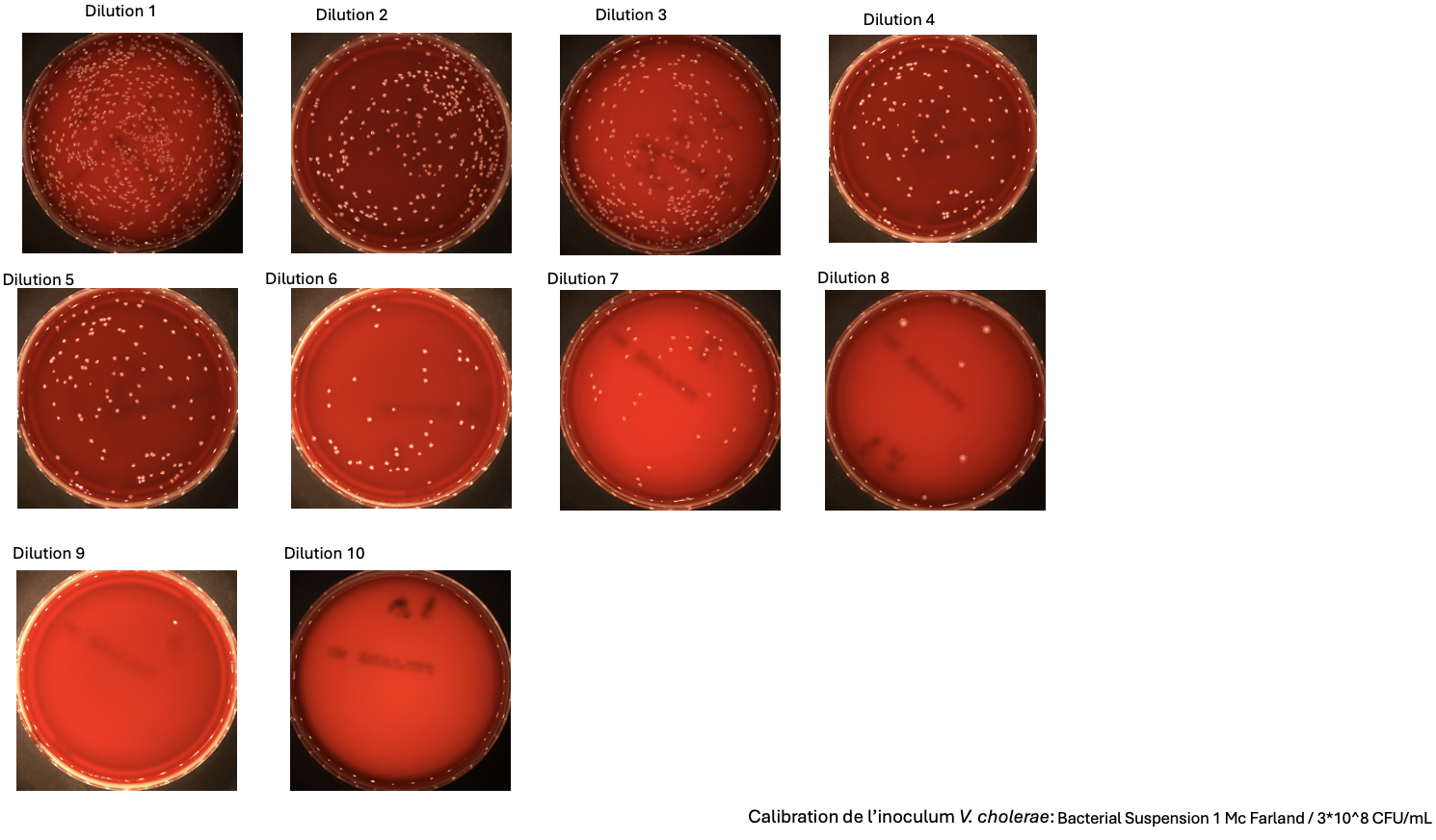


**Figure S7:** bacterial suspensions of 1 McFarland density (≈ 3 × 10⁸ cells/mL) were prepared, then **subjected to successive decimal dilutions.** Each dilution was **inoculated on Columbia blood agar (COS)** and incubated at 37 °C for 24 h for enumeration.
