# Supplementary material for: Isothermal amplification and colorimetric detection of Vibrio cholerae in environmental matrices
Source: Microbiol Spectr. 2026 May 21;14(7):e02753-25. doi: 10.1128/spectrum.02753-25 (PMC13340027; doi:10.1128/spectrum.02753-25)
Supplement: Reviewer comments [file reviewer-comments.pdf]

AMERICAN SOCIETY FOR MICROBIOLOGY | Microbiology Spectrum

Isothermal amplification and colorimetric detection of Vibrio cholerae in environmental matrices.

Michelande Adolphe, Mahmoud Boualam, Hamadou Oumarou Hama, and Elodie Terrer

Corresponding Author(s): Elodie Terrer, Aix-Marseille Universite

Review Timeline:

Submission Date:	September 9, 2025
Editorial Decision:	October 24, 2025
Revision Received:	December 19, 2025
Accepted:	March 5, 2026

Editor: Salina Parveen

Reviewer(s): Disclosure of reviewer identity is with reference to reviewer comments included in decision letter(s). The following individuals involved in review of your submission have agreed to reveal their identity: Max Maurin (Reviewer #1); Ghiles Grine (Reviewer #2)

Transaction Report:

(Note: With the exception of the correction of typographical or spelling errors that could be a source of ambiguity, letters and reports are not edited. The original formatting of letters and referee reports may not be reflected in this compilation.)

DOI: https://doi.org/10.1128/spectrum.02753-25

Re: Spectrum02753-25 (Isothermal amplification and colorimetric detection of Vibrio cholerae in environmental matrices.)

Dear Prof. Elodie Terrer:

Thank you for the privilege of reviewing your work. Below you will find my comments, instructions from the Spectrum editorial office, and the reviewer comments.

This manuscript presents a practical LAMP-based protocol for rapid detection of Vibrio cholerae in environmental samples using alkaline lysis extraction and colorimetric detection. However, several aspects require clarification and improvement before publication, especially experimental design, conclusions, specificity and sensitivity of the testing methods.

Please return the manuscript within 60 days; if you cannot complete the modification within this time period, please contact me. If you do not wish to modify the manuscript and prefer to submit it to another journal, notify me immediately so that the manuscript may be formally withdrawn from consideration by Spectrum.

Revision Guidelines
To submit your modified manuscript, log into the submission site at https://spectrum.msubmit.net/cgi-bin/main.plex. Go to Author Tasks and click the appropriate manuscript title to begin. The information you entered when you first submitted the paper will be displayed; update this as necessary. Note the following requirements:

• Upload point-by-point responses to the issues raised by the reviewers in a file named "Response to Reviewers," NOT in your cover letter.
• Upload a compare copy of the manuscript (without figures) as a "Marked-Up Manuscript" file.
• Upload a clean .DOC/.DOCX version of the revised manuscript and remove the previous version.
• Each figure must be uploaded as a separate, editable, high-resolution file (TIFF or EPS preferred), and any multipanel figures must be assembled into one file.
• Any supplemental material intended for posting by ASM should be uploaded with their legends separate from the main manuscript. You can combine all supplemental material into one file (preferred) or split it into a maximum of 10 files with all associated legends included.

For complete guidelines on revision requirements, see our Submission and Review Process webpage. Submission of a paper that does not conform to guidelines may delay acceptance of your manuscript.

Data availability: ASM policy requires that data be available to the public upon online posting of the article, so please verify all links to sequence records, if present, and make sure that each number retrieves the full record of the data. If a new accession number is not linked or a link is broken, provide Spectrum production staff with the correct URL for the record. If the accession numbers for new data are not publicly accessible before the expected online posting of the article, publication may be delayed; please contact production staff (Spectrum@asmusa.org) immediately with the expected release date.

Publication Fees: For information on publication fees and which article types are subject to charges, visit our website. If your manuscript is accepted for publication and any fees apply, you will be contacted separately about payment during the production process; please follow the instructions in that e-mail. Arrangements for payment must be made before your article is published.

ASM Membership: Corresponding authors may join or renew ASM membership to obtain discounts on publication fees. Need to upgrade your membership level? Please contact Customer Service at Service@asmusa.org.

The ASM Journals program strives for constant improvement in our submission and publication process. Please tell us how we can improve your experience by taking this quick Author Survey.

Thank you for submitting your paper to Spectrum.

Sincerely,
Salina Parveen
Editor
Microbiology Spectrum

Reviewer #1 (Comments for the Author):

The manuscript describes the development and evaluation of an isothermal amplification assay for detection of Vibrio cholerae in environmental samples.

Major Comments.
Given the existence of twelve pathogenic Vibrio species, numerous non-pathogenic Vibrio species, and a wide range of other pathogens capable of causing diarrhea in humans, the developed test must be not only highly sensitive but, more importantly, highly specific to ensure accurate detection of Vibrio cholerae in environmental samples. This requirement is critically important for implementing appropriate prophylactic measures in cholera-endemic areas.
A major limitation of the study is that only four V. cholerae strains, three other Vibrio species, and more significantly only four non-Vibrio species were tested. This limited scope is insufficient for a robust evaluation of the test's sensitivity and specificity. Moreover, the selected non-Vibrio species are not the most relevant; this group should have included human enteric pathogens other than Vibrio.
Other Comments.
The abstract mentions in line 26 that "The sensitivity of 1000 cells/mL on four V. cholerae isolates fell to 3*10 8 cells/mL after V. cholerae was incorporated into five mock infected environmental matrixes (soil, sand, 27 seawater, tap water and anthropized effluents), yielding 100% positivity". This sentence is unclear. Does it mean that the sensitivity of the isothermal amplification assay dropped to 3×10^8 cells/mL when V. cholerae was incorporated into these environmental matrices? If so, this represents very poor sensitivity and may not be suitable for field investigations.
Page 6, line 100 and the corresponding paragraph in the Materials and Methods section: the authors should provide more detailed information about the SYBR PCR. What was the gene target? What primers and PCR cycling conditions were used? What controls were implemented to evaluate the quality of DNA extraction from the samples?
Page 7, line 120: Control matrices were spiked with 10^4 CFU/mL of bacteria. Throughout the manuscript, CFU counts are evaluated solely based on McFarland turbidity measurements. The actual bacterial inoculum should have been verified using a CFU culture method. McFarland standards are not always accurate and results may vary depending on the bacterial species tested. Besides, testing several spiked bacterial inocula would have been more relevant to isothermal amplification assay performances.
Discussion section, line 157: The authors cannot claim that "This study validates the effectiveness of the LAMP method for the rapid detection and control of V. cholerae in the environment" since no field study under real-life conditions was conducted.
Conclusion The study is too preliminary to support the claim that low-income countries could benefit from this new V. cholerae isothermal amplification assay for improving prophylaxis and management of cholera cases. Additionally, information about the expected cost of the test would be valuable for readers.
Minor Comments
Importance section, line 37: The phrase "epidemic remains endemic" is unclear and should be clarified.
Page 5, line 76: The sentence ending with "and acute" appears incomplete.

Reviewer #2 (Comments for the Author):

This manuscript presents a practical LAMP-based protocol for rapid detection of Vibrio cholerae in environmental samples using alkaline lysis extraction and colorimetric detection. The work addresses an important public health need for point-of-care detection in cholera-endemic regions. The methodology is sound and the results are promising. However, several aspects require clarification and improvement before publication.

1- Line 26-27: The manuscript reports a dramatic loss of sensitivity from 10^3 cells/mL (pure culture) to 3×10^8 cells/mL in environmental matrices. This represents a 5-log reduction in sensitivity, which is extremely concerning and severely limits practical application.
This sensitivity (3×10^8 cells/mL = 300 million cells/mL) is far too high for meaningful environmental monitoring
The text states "100% positivity" but this is only at an unrealistically high concentration
This critical limitation is not adequately discussed or explained
Is this a typo? Should it be 3×10^3 cells/mL instead of 3×10^8?
Clarify if this is a typographical error
If correct, provide detailed discussion of why sensitivity drops so dramatically
Discuss whether this level of sensitivity is adequate for real-world applications
Consider optimization experiments to improve sensitivity in environmental matrices
Compare with sensitivities reported in other LAMP studies for V. cholerae
2- All experiments use "mock-contaminated" (spiked) samples, not real environmental samples from cholera-endemic areas.
Spiked samples don't reflect the complexity of real samples (competing microbiota, PCR inhibitors, degraded DNA)
The system may perform very differently with naturally occurring V. cholerae
Environmental V. cholerae may be in VBNC (viable but non-culturable) state or within biofilms.
Required from the authors: Acknowledge this as a major limitation in the Discussion
Discuss plans for validation with authentic environmental samples
Address how biofilms or VBNC cells might affect detection
Consider testing at least a few archived environmental samples if available.

3- DNA Extraction (Lines 187-197):

What was the final volume after neutralization? (100 + 100 + 100 = 300 µL?)
Was this used directly or further diluted?
Storage conditions for extracted DNA not specified
4- LAMP Protocol (Lines 212-228):
How was the primer mix prepared? (concentrations in stock solution)
The math for primer dilution (line 216-218) is confusing and hard to follow
Was each reaction performed in duplicate or triplicate? Not stated

5-Environmental Matrices (Lines 229-238):

Source/origin of environmental samples not specified (where exactly?)
Were samples sterilized before spiking? (important for specificity claims)
Soil and sand processing: was centrifugation sufficient to remove inhibitors?
Final volume after processing not clear
6- Lines 125-130 & Figure S6: RT-PCR SYBR was performed as a control but results are relegated to supplementary materials.
Move RT-PCR comparison to main Results section
Create a comparison table: LAMP vs RT-PCR for each matrix
Discuss advantages/disadvantages of each method
7- Discussion lacks depth
Expand Discussion to ~2-3 pages
Add detailed comparison with other detection methods (Table format recommended)
Discuss practical deployment: equipment needed, training requirements, cost per test
Address limitations more thoroughly
Discuss next steps for validation and implementation

December 15th, 2025

Dear Editor,

Please find enclosed the revised version of our manuscript Spectrum02753-25 entitled "Isothermal amplification and colorimetric detection of *Vibrio cholerae* in environmental matrices." along with the answers of the authors to the Reviewers' comments.

Reviewer #1 (Comments for the Author):

The manuscript describes the development and evaluation of an isothermal amplification assay for detection of *Vibrio cholerae* in environmental samples.

Major Comments.

Given the existence of twelve pathogenic Vibrio species, numerous non-pathogenic Vibrio species, and a wide range of other pathogens capable of causing diarrhea in humans, the developed test must be not only highly sensitive but, more importantly, highly specific to ensure accurate detection of *Vibrio cholerae* in environmental samples. This requirement is critically important for implementing appropriate prophylactic measures in cholera-endemic areas. A major limitation of the study is that only four *V. cholerae* strains, three other Vibrio species, and more significantly only four non-Vibrio species were tested. This limited scope is insufficient for a robust evaluation of the test's sensitivity and specificity. Moreover, the selected non-Vibrio species are not the most relevant; this group should have included human enteric pathogens other than Vibrio.

Authors 'answer: The reviewer is perfectly right; this section has been completed in the revised version.

We fully share the importance of the issue raised, namely the robust specificity of the detection system regarding *Vibrio cholerae*, in a context where many species of *Vibrio* and other enteric pathogens can be present simultaneously in aquatic and environmental environments. This choice was mainly aimed at validating the primary specificity of the system, i.e. its ability to distinguish *V. cholerae* from phylogenetically related *Vibrio* and from a representative group of unrelated enteric bacteria.

Lines 132-136: Before DNA extraction, samples were not decontaminated or sterilized prior to inoculation, to preserve microbial diversity and inhibitor loading typical of environmental matrices. This methodological choice aimed to test the robustness and specificity of the LAMP system in more representative field conditions, rather than in an artificially purified environment.

Line 232-234: And the blastn *in silico* of the primers shows a hook only with *V. cholerae* as indicated in the Materials and Methods section and supplementary data have been added (**Figure S6**).

Other Comments.

The abstract mentions in line 26 that "The sensitivity of 1000 cells/mL on four V. cholerae isolates fell to 3*10 8 cells/mL after V. cholerae was incorporated into five mock infected environmental matrixes (soil, sand, 27 seawater, tap water and anthropized effluents), yielding 100% positivity". This sentence is unclear. Does it mean that the sensitivity of the isothermal amplification assay dropped to 3×10^8 cells/mL when V. cholerae was incorporated into these environmental matrices? If so, this represents very poor sensitivity and may not be suitable for field investigations.

Authors 'answer: The reviewer is perfectly right; a clarification has been included in the revised version.

The bacterial inoculum was a 3×10^8 cell/mL suspension (1 McFarland). From this suspension, serial dilutions were performed to evaluate the analytical detection limit of the LAMP assay ranging from 10^8–10^0 dilutions. For environmental simulations, *V. cholerae at* 10^4 dilution was incorporated into the different matrices (soil, sand, seawater, tap water and anthropogenic effluents). This deliberately low concentration was intended to evaluate the system's ability to detect the bacterium in complex and naturally inhibitory environments, while at the same time approaching the microbial loads that could be encountered in contaminated environmental samples. Thus, the value of 3×10^8 cells/mL does not represent a loss of sensitivity, but the starting concentration used for dilutions.

Lines 23-27: A positive signal was detected across a range of 3×10^8 to 3×10^3 cells/mL for four *V. cholerae* isolates within 30–60 minutes. Following the inoculation of 3×10^4 *V. cholerae* cells into five simulated environmental matrices (soil, sand, seawater, tap

water, and anthropogenic effluents), positive results were similarly obtained between $3{\times}10^4$ and $3{\times}10^3$ cells/mL over the same time interval.

Page 6, line 100 and the corresponding paragraph in the Materials and Methods section: the authors should provide more detailed information about the SYBR PCR. What was the gene target? What primers and PCR cycling conditions were used? What controls were implemented to evaluate the quality of DNA extraction from the samples?

Authors'answer: The reviewer is perfectly right; this section has been completed in the revised version.

Lines 236: For amplification F3-B3 outer primers loop primers targeting *V. cholerae* *rpoB* gene.

Lines 240-243: A DNA-free mixture was used as a negative control. Amplification consisting in 10-minute polymerase activation at 95° C followed by 39 cycles of 10-second denaturation at 95° C and 30-second hybridization at 60° C, was performed on a BIO-RAD T100TM Thermal Cycler thermocycler (Bio-Rad Inc., Hercules, CA).

Page 7, line 120: Control matrices were spiked with 10^4 CFU/mL of bacteria. Throughout the manuscript, CFU counts are evaluated solely based on McFarland turbidity measurements. The actual bacterial inoculum should have been verified using a CFU culture method. McFarland standards are not always accurate and results may vary depending on the bacterial species tested. Besides, testing several spiked bacterial inocula would have been more relevant to isothermal amplification assay performances.

Authors'answer: The reviewer is perfectly right; this section has been completed in the revised version.

Lines 269-275: After turbidity adjustment using the McFarland standard, the corresponding concentration was verified by culture-based enumeration. Bacterial suspensions adjusted to 1 McFarland unit ($\approx 3 \times 10^8$ cells/mL) were prepared and subjected to successive tenfold dilutions (10^7 to 10^1). Each dilution was plated on Columbia blood agar (COS) and incubated at 37 °C for 24 h to determine colony-forming units (CFU), thereby confirming the agreement between the McFarland estimation and the actual bacterial concentration (Supplementary Data: Figure S7).

Discussion section, line 157: The authors cannot claim that "This study validates the effectiveness of the LAMP method for the rapid detection and control of *V. cholerae* in the environment" since no field study under real-life conditions was conducted.

Authors'answer: The reviewer is perfectly right; this section has been completed in the revised version.

Line 174-182: This study presents the development of a LAMP system for the rapid detection of *Vibrio cholerae* in different environmental matrices (seawater, sand, soil, tap water and anthropogenic effluents). The results demonstrate **the** feasibility and analytical specificity of the method, with a detection limit adapted to environmental monitoring applications, and it is precisely for this reason that, in the present study, the samples were not treated or decontaminated, to assess the intrinsic specificity of the system under conditions as close as possible to environmental reality. While promising, the method would still require field validation, under real-world environmental conditions, to confirm operational performance and reliability at scale.

Conclusion The study is too preliminary to support the claim that low-income countries could benefit from this new V. cholerae isothermal amplification assay for improving prophylaxis and management of cholera cases. Additionally, information about the expected cost of the test would be valuable for readers.

Authors'answer: The reviewer is perfectly right; this section has been completed in the revised version.

Lines 185-186: and the cost per test could be estimated at £2.66 (21).

Lines 191-195: This study is a proof of concept demonstrating the analytical feasibility and specificity of the LAMP system for the rapid detection of *Vibrio cholerae* in different environmental matrices. The results obtained confirm a potential approach for future applications in environmental monitoring and early detection of cholera.

Minor Comments

Importance section, line 37: The phrase "epidemic remains endemic" is unclear and should be clarified.

Authors'answer: The reviewer is perfectly right; this section has been completed in the revised version.

Lines 36-38: Although various detection tests have been developed for different types of samples, **cholera** remains endemic in several countries**,** particularly where access to safe drinking water is limited and sanitation infrastructure remains inadequate.

Page 5, line 76: The sentence ending with "and acute" appears incomplete.

Authors'answer: The reviewer is perfectly right; this section has been completed in the revised version.

Line 80-82: In October 2024, a total of 37 363 new cases of cholera and acute diarrhea were reported in 19 countries across three WHO regions (African Region, Eastern Mediterranean Region and South-East Asian region)

Reviewer #2 (Comments for the Author)

This manuscript presents a practical LAMP-based protocol for rapid detection of Vibrio cholerae in environmental samples using alkaline lysis extraction and colorimetric detection. The work addresses an important public health need for point-of-care detection in cholera-endemic regions. The methodology is sound and the results are promising. However, several aspects require clarification and improvement before publication.

1- Line 26-27: The manuscript reports a dramatic loss of sensitivity from 10^3 cells/mL (pure culture) to 3×10^8 cells/mL in environmental matrices. This represents a 5-log reduction in sensitivity, which is extremely concerning and severely limits practical application.

This sensitivity (3×10^8 cells/mL = 300 million cells/mL) is far too high for meaningful environmental monitoring

The text states "100% positivity" but this is only at an unrealistically high concentration

This critical limitation is not adequately discussed or explained

Is this a typo? Should it be 3×10^3 cells/mL instead of 3×10^8?

Clarify if this is a typographical error

If correct, provide detailed discussion of why sensitivity drops so dramatically

Discuss whether this level of sensitivity is adequate for real-world applications

Consider optimization experiments to improve sensitivity in environmental matrices

Compare with sensitivities reported in other LAMP studies for V. cholerae

Authors'answer: The reviewer is perfectly right; the same point was raised by the first reviewer, and it has been addressed and clarified in the revised version.

Lines 23-27: A positive signal was detected across a range of $3×10^8$ to $3×10^3$ cells/mL for four *V. cholerae* isolates within 30–60 minutes. Following the inoculation of $3×10^4$ *V. cholerae* cells into five simulated environmental matrices (soil, sand, seawater, tap water, and anthropogenic effluents), positive results were similarly obtained between $3×10^4$ and $3×10^3$ cells/mL over the same time interval.

2- All experiments use "mock-contaminated" (spiked) samples, not real environmental samples from cholera-endemic areas. Spiked samples don't reflect the complexity of real samples (competing microbiota, PCR inhibitors, degraded DNA)

The system may perform very differently with naturally occurring V. cholerae

Environmental V. cholerae may be in VBNC (viable but non-culturable) state or within biofilms.

Required from the authors: Acknowledge this as a major limitation in the Discussion

Discuss plans for validation with authentic environmental samples

Address how biofilms or VBNC cells might affect detection

Consider testing at least a few archived environmental samples if available.

Authors'answer: The reviewer is perfectly right; this section has been completed in the revised version.

We fully share the idea that the behavior of the system could differ when applied to real, more complex, and potentially inhibitor-rich environmental samples.

Lines 262-264: None of these samples were sterilized or decontaminated prior to seeding (*spiking*) with *Vibrio cholerae*, to evaluate the performance of the system under near-field environmental conditions.

3- DNA Extraction (Lines 187-197):

What was the final volume after neutralization? (100 + 100 + 100 = 300 µL?)

Was this used directly or further diluted?

Storage conditions for extracted DNA not specified

Authors 'answer: The reviewer is perfectly right; this section has been completed in the revised version.

Line 221-223: The resulting final volume of 300 µL was partially used for subsequent procedures, while the remaining aliquots were stored at –20 °C until use.

4- LAMP Protocol (Lines 212-228):
How was the primer mix prepared? (concentrations in stock solution)
The math for primer dilution (line 216-218) is confusing and hard to follow
Was each reaction performed in duplicate or triplicate? Not stated

Authors 'answer: The reviewer is perfectly right; this section has been completed in the revised version.

Lines 245-249: Primers were prepared from a 1000 µM stock solution. Each 50 µL reaction contained 16 µmol/L of inner primers (FIB and BIP), 2 µmol/L of outer primers (F3 and B3), 4 µmol/L of loop primers (LF and LB), 44 µL of water (Invitrogen, Paisley, UK) resuspended in the RT-LAMP colorimetric master mix (Biotechnology Co, Wuhan, Hubei, China) and 1 µL of DNA.

Lines 280-283: Each reaction was performed in triplicate, by different operators who received the same protocol, the same reactants and the same equipment. The independent replicates all yielded similar results, confirming the reproducibility and robustness of the LAMP system used.

5-Environmental Matrices (Lines 229-238):

Source/origin of environmental samples not specified (where exactly?)
Were samples sterilized before spiking? (important for specificity claims)
Soil and sand processing: was centrifugation sufficient to remove inhibitors?
Final volume after processing not clear

Authors 'answer: The reviewer is perfectly right; this section has been completed in the revised version.

The samples were centrifuged to separate the pellets. This centrifugation step probably contributed to the elimination of some inhibitors, as suggested by the results obtained in comparison with negative controls.

Line 261: A total of five environmental matrixes in Marseille were used: soil, sand, seawater, tap water, and anthropogenic effluents.

Lines 261-264: A total of five environmental matrixes in Marseille were used: soil, sand, seawater, tap water, and anthropogenic effluents. None of these samples were sterilized or decontaminated prior to seeding (*spiking*) with *Vibrio cholerae*, to evaluate the performance of the system under near-field environmental conditions.

Lines 275-280: For each matrix, 100 µL were spiked with 100 µL of a 10^4 CFU/mL *V. cholerae* suspension and, in parallel, with 100 µL of a 10^4 CFU/mL non-cholera *Vibrio* suspension. From each inoculated matrix, 100 µL were collected for DNA extraction using the alkaline lysis protocol. One microliter of the resulting 300 µL DNA extract was incorporated into the LAMP-*V. cholerae* assay and rt-PCR.

Lines 132-136: Before DNA extraction, samples were not decontaminated or sterilized prior to inoculation, to preserve microbial diversity and inhibitor loading typical of environmental matrices. This methodological choice aimed to test the robustness and specificity of the LAMP system in more representative field conditions, rather than in an artificially purified environment.

6- Lines 125-130 & Figure S6: RT-PCR SYBR was performed as a control, but results are relegated to supplementary materials.

Move RT-PCR comparison to main Results section

Create a comparison table: LAMP vs RT-PCR for each matrix

Discuss advantages/disadvantages of each method

Authors 'answer: The reviewer is perfectly right; these observations and suggestions have been fully addressed in the revised version.

Lines 406-413:

Table 2. LAMP-*V. cholerae* shows a constant detection limit (10^4 copies) for all samples, confirming its specificity under various environmental conditions; RT-PCR has high Ct (> 34), which indicates a low amount of target DNA and possible **inhibition** due to the template; tap water and effluents offer the best performance (few inhibitors); soil, sand and sea water show inhibition effects related to the chemical and organic complexity of the samples, the LAMP method is more tolerant and fast, but RT-PCR remains more accurate for fine quantification.

Comparison table LAMP-*V.cholerae* & RT-PCR-*V.cholerae*					Comparative Methods	
Sample	10^4	10^3	10^2	10^1	LAMP	RT-PCR
Tap water	LAMP(+) & RT-PCR (+)	LAMP(-) & RT-PCR (+)	LAMP(-) & RT-PCR (+)	LAMP(-) & RT-PCR (+)	Detection limit 10^4	Ct = 32.93 to 10^1
Effluents	LAMP(+) & RT-PCR (+)	LAMP(-) & RT-PCR (+)	LAMP(-) & RT-PCR (+)	LAMP(-) & RT-PCR (+)	Detection limit 10^4	Ct = 34.28 to 10^1
Soil	LAMP(+) & RT-PCR (-)	LAMP(-) & RT-PCR (-)	LAMP(-) & RT-PCR (-)	LAMP(-) & RT-PCR (-)	Detection limit 10^4	Ct = 36.09 to 10^4
Sea water	LAMP(+) & RT-PCR (-)	LAMP(-) & RT-PCR (-)	LAMP(-) & RT-PCR (-)	LAMP(-) & RT-PCR (-)	Detection limit 10^4	Ct = 36.43 to 10^4
Sand	LAMP(+) & RT-PCR (+)	LAMP(-) & RT-PCR (+)	LAMP(-) & RT-PCR (-)	LAMP(-) & RT-PCR (-)	Detection limit 10^4	Ct = 36.43 to 10^2

7- Discussion lacks depth

Expand Discussion to ~2-3 pages

Add detailed comparison with other detection methods (Table format recommended)

Discuss practical deployment: equipment needed, training requirements, cost per test

Address limitations more thoroughly

Discuss next steps for validation and implementation

Authors 'answer: The reviewer is perfectly right; this section has been completed in the revised version.

Line 414-417: Table 3. Overview of *Vibrio cholerae* detection methods, detailing their analytical principles, sample types, indicative lead times, result quality, key strengths, and major limitations.

Method	Principle	Samples	Incative Lead Times	Quality	Key Strengths	Limites	References
Culture	APW enrichment and selective isolation on TCBS	Stool, Them, Food, Surface Water	24-72 H	Moderate sensitivity; High specificity	"Gold standard" for possible confirmation, antibiogram and genotyping	Lack of sensitivity under low load or VBNC status	**(Error! Reference source not found.)**
PCR	Amplificaation ADN	Water, sediment, complex matrices	4-8 H	Sensitivity/Specificity Good (depends on primers)	Simple, inexpensive, multiplex possible	Not very quantitative; Sensitive to inhibitors	**(Error! Reference source not found.)**
qPCR(real-time)	Quantitative detection	Water (tap/sea/waste)	2-3 H	High sensitivity & specificity; quantifiable	Speed, quantification, multiplex	dead cell DNA; matrix inhibition	**(Error! Reference source not found.)**
LAMP	Isothermal amplification (60-67° Celcieus) targets specific gene	Water, sediment	30-60 min	High sensitivity; fast	Very suitable for the terrain**;** Inhibitor tolerance; visual readout/fluorescence	Contamination; qualitative non-quantitative; False positive	**(Error! Reference source not found.)**
Immunological test	Immunochromatography	Stool (clinical)	15-30 min	Sensitivity/Specificity varies according to batch & context	Very fast field screening	Variable return: Culture/qPCR confirmation required	**(Error! Reference source not found.)**
ELISA (toxin/antigen)	Antigen detection	Stool	2-5 H	Correct sensitivity; specific to the targeted antigen	Standardized, multi-well	Antigen specificity; less useful under very low load	**(Error! Reference source not found.)**

The implementation of a comprehensive comparison and operational evaluation of the field deployment would be a project, requiring additional experimentation, consolidated cost data and multicenter validations. The main objective was to demonstrate the feasibility and analytical performance of the LAMP method in an environmental screening context.

Line 144-159: However, these observations obviously do not guarantee that un-mastered field conditions including parameters such as physical and chemical

characteristics of environmental samples such as temperature, acidity, potential inhibitors would not influence the limit of detection. Some limitations must be recognized, the trials were carried out under controlled experimental conditions and on a limited number of *V. cholerae* strains and non-target species, which does not yet allow a full evaluation of the specificity of the test in the face of environmental bacterial diversity**.** The presence of natural inhibitors (organic matter, salts, clays, etc.) in the actual samples could affect the effective sensitivity of the test, despite the performance observed in the laboratory.

The LAMP method, although extremely sensitive, does not distinguish between viable and non-viable bacteria DNA, which can lead to an overestimation of environmental risk. To obtain results that are close to the field, the environmental samples were used in their raw state without treatment or decontamination. Validation in real field conditions, on a wider panel of samples and isolates from endemic areas, will be required to confirm the robustness, specificity and transferability of the system.

Supplementary figure S6: Screenshot NCBI Blastn *in silico* of the primers.

Distance tree of results MSA viewer

Descriptions | Graphic Summary | Alignments | Taxonomy

Sequences producing significant alignments — Download — Select columns — Show 100

select all *100 sequences selected* — GenBank Graphics Distance tree of results MSA Viewer

Description	Scientific Name	Max Score	Total Score	Query Cover	E value	Per. Ident	Acc. Len	Accession
Vibrio cholerae strain 105Vc10 chromosome 1, complete sequence	Vibrio cholerae	44.1	44.1	100%	0.10	100.00%	3065653	CP189154.1
Vibrio cholerae strain 111Vc07 chromosome 1, complete sequence	Vibrio cholerae	44.1	44.1	100%	0.10	100.00%	3057147	CP189277.1
Vibrio cholerae strain 123Vc05 chromosome 1, complete sequence	Vibrio cholerae	44.1	44.1	100%	0.10	100.00%	3093912	CP189041.1
Vibrio cholerae strain 104Vc05 chromosome 1, complete sequence	Vibrio cholerae	44.1	44.1	100%	0.10	100.00%	3065663	CP189178.1
Vibrio cholerae strain 114Vc08 chromosome, complete genome	Vibrio cholerae	44.1	44.1	100%	0.10	100.00%	4136123	CP189118.1
Vibrio cholerae strain 105Vc06 chromosome 1, complete sequence	Vibrio cholerae	44.1	44.1	100%	0.10	100.00%	3063268	CP189162.1
Vibrio cholerae strain 108Vc06 chromosome 1, complete sequence	Vibrio cholerae	44.1	44.1	100%	0.10	100.00%	3065661	CP189336.1
Vibrio cholerae strain 112Vc04 chromosome, complete genome	Vibrio cholerae	44.1	44.1	100%	0.10	100.00%	4136125	CP189267.1
Vibrio cholerae strain 129Vc02 chromosome 1, complete sequence	Vibrio cholerae	44.1	44.1	100%	0.10	100.00%	3058023	CP188991.1
Vibrio cholerae strain 110Vc03 chromosome 1, complete sequence	Vibrio cholerae	44.1	44.1	100%	0.10	100.00%	3057135	CP189302.1
Vibrio cholerae strain 125Vc04 chromosome 1, complete sequence	Vibrio cholerae	44.1	44.1	100%	0.10	100.00%	3063772	CP189017.1
Vibrio cholerae strain 114Vc02 chromosome 1, complete sequence	Vibrio cholerae	44.1	44.1	100%	0.10	100.00%	3058925	CP189126.1
Vibrio cholerae strain 119Vc01 chromosome	Vibrio cholerae	44.1	44.1	100%	0.10	100.00%	4136449	CP189109.1
Vibrio cholerae strain 134Vc06 chromosome 1, complete sequence	Vibrio cholerae	44.1	44.1	100%	0.10	100.00%	3058908	CP188910.1
Vibrio cholerae strain 105Vc08 chromosome 1, complete sequence	Vibrio cholerae	44.1	44.1	100%	0.10	100.00%	3063271	CP189158.1
Vibrio cholerae strain 104Vc03 chromosome 1, complete sequence	Vibrio cholerae	44.1	44.1	100%	0.10	100.00%	3065663	CP189182.1
Vibrio cholerae strain 105Vc03 chromosome 1, complete sequence	Vibrio cholerae	44.1	44.1	100%	0.10	100.00%	3063270	CP189166.1
Vibrio cholerae strain 124Vc02 chromosome 1, complete sequence	Vibrio cholerae	44.1	44.1	100%	0.10	100.00%	3063770	CP189029.1
Vibrio cholerae strain 122Vc02 chromosome 1, complete sequence	Vibrio cholerae	44.1	44.1	100%	0.10	100.00%	3093912	CP189067.1
Vibrio cholerae strain 121Vc07 chromosome, complete genome	Vibrio cholerae	44.1	44.1	100%	0.10	100.00%	4134359	CP189076.1

Job Title

LB (rpoB)

RID

H672UEBH014 *Search expires on 11-12 18:22 pm*

Download All

Program

BLASTN Citation

Database

core_nt See details

Query ID

lcl|Query_5211083

Description

None

Molecule type

dna

Query Length

25

Other reports

Distance tree of results MSA viewer

Filter Results

Organism *only top 20 will appear* — exclude

Type common name, binomial, taxid or group name

+ Add organism

Percent Identity to — **E value** to — **Query Coverage** to

Filter — Reset

Descriptions | Graphic Summary | Alignments | Taxonomy

Sequences producing significant alignments

Download | Select columns | Show 100

select all *100 sequences selected*

GenBank | Graphics | Distance tree of results | MSA Viewer

Description	Scientific Name	Max Score	Total Score	Query Cover	E value	Per. Ident	Acc. Len	Accession
Vibrio cholerae strain 105Vc10 chromosome 1, complete sequence	Vibrio cholerae	50.1	50.1	100%	0.003	100.00%	3065653	CP189154.1
Vibrio cholerae strain 111Vc07 chromosome 1, complete sequence	Vibrio cholerae	50.1	50.1	100%	0.003	100.00%	3057147	CP189277.1
Vibrio cholerae strain 123Vc05 chromosome 1, complete sequence	Vibrio cholerae	50.1	50.1	100%	0.003	100.00%	3093912	CP189041.1
Vibrio cholerae strain 104Vc05 chromosome 1, complete sequence	Vibrio cholerae	50.1	50.1	100%	0.003	100.00%	3065663	CP189178.1
Vibrio cholerae strain 114Vc08 chromosome, complete genome	Vibrio cholerae	50.1	50.1	100%	0.003	100.00%	4136123	CP189118.1
Vibrio cholerae strain 113Vc02 chromosome, complete genome	Vibrio cholerae	50.1	50.1	100%	0.003	100.00%	4137888	CP189138.1
Vibrio cholerae strain 114Vc06 chromosome, complete genome	Vibrio cholerae	50.1	50.1	100%	0.003	100.00%	4136127	CP189120.1
Vibrio cholerae strain 105Vc06 chromosome 1, complete sequence	Vibrio cholerae	50.1	50.1	100%	0.003	100.00%	3063268	CP189162.1
Vibrio cholerae strain 108Vc06 chromosome 1, complete sequence	Vibrio cholerae	50.1	50.1	100%	0.003	100.00%	3065661	CP189336.1
Vibrio cholerae strain 109Vc10 chromosome 1, complete sequence	Vibrio cholerae	50.1	50.1	100%	0.003	100.00%	3065660	CP189308.1
Vibrio cholerae strain 112Vc04 chromosome, complete genome	Vibrio cholerae	50.1	50.1	100%	0.003	100.00%	4136125	CP189267.1
Vibrio cholerae strain 110Vc03 chromosome 1, complete sequence	Vibrio cholerae	50.1	50.1	100%	0.003	100.00%	3057135	CP189302.1
Vibrio cholerae strain 125Vc04 chromosome 1, complete sequence	Vibrio cholerae	50.1	50.1	100%	0.003	100.00%	3063772	CP189017.1
Vibrio cholerae strain 114Vc02 chromosome 1, complete sequence	Vibrio cholerae	50.1	50.1	100%	0.003	100.00%	3058925	CP189126.1
Vibrio cholerae strain 119Vc01 chromosome	Vibrio cholerae	50.1	50.1	100%	0.003	100.00%	4136449	CP189109.1
Vibrio cholerae strain 105Vc03 chromosome 1, complete sequence	Vibrio cholerae	50.1	50.1	100%	0.003	100.00%	3063270	CP189166.1
Vibrio cholerae strain 124Vc02 chromosome 1, complete sequence	Vibrio cholerae	50.1	50.1	100%	0.003	100.00%	3063770	CP189029.1
Vibrio cholerae strain 110Vc02 chromosome 1, complete sequence	Vibrio cholerae	50.1	50.1	100%	0.003	100.00%	3057144	CP189304.1
Vibrio cholerae strain 122Vc02 chromosome 1, complete sequence	Vibrio cholerae	50.1	50.1	100%	0.003	100.00%	3093912	CP189067.1
Vibrio cholerae strain 121Vc07 chromosome, complete genome	Vibrio cholerae	50.1	50.1	100%	0.003	100.00%	4134359	CP189076.1
Vibrio cholerae strain 121Vc03 chromosome 1, complete sequence	Vibrio cholerae	50.1	50.1	100%	0.003	100.00%	3057144	CP189081.1

Feedback

Job Title

F3 (rpoB)

RID

H678F6E6014 *Search expires on 11-12 18:25 pm*

Download All

Program

BLASTN Citation

Database

core_nt See details

Query ID

lcl|Query_5368093

Description

None

Molecule type

nucleic acid

Query Length

19

Other reports

Distance tree of results MSA viewer

Filter Results

Organism *only top 20 will appear* exclude

Type common name, binomial, taxid or group name

+ Add organism

Percent Identity to **E value** to **Query Coverage** to

Filter Reset

Descriptions | Graphic Summary | Alignments | Taxonomy

Sequences producing significant alignments Download Select columns Show 100

select all 100 sequences selected GenBank Graphics Distance tree of results MSA Viewer

Description	Scientific Name	Max Score	Total Score	Query Cover	E value	Per. Ident	Acc. Len	Accession
Vibrio cholerae strain 105Vc10 chromosome 1, complete sequence	Vibrio cholerae	38.2	38.2	100%	6.2	100.00%	3065653	CP189154.1
Vibrio cholerae strain 111Vc07 chromosome 1, complete sequence	Vibrio cholerae	38.2	38.2	100%	6.2	100.00%	3057147	CP189277.1
Vibrio cholerae strain 134Vc10 chromosome 1, complete sequence	Vibrio cholerae	38.2	38.2	100%	6.2	100.00%	3058911	CP188904.1
Vibrio cholerae strain 135Vc06 chromosome, complete genome	Vibrio cholerae	38.2	38.2	100%	6.2	100.00%	4138010	CP188901.1
Vibrio cholerae strain 104Vc05 chromosome 1, complete sequence	Vibrio cholerae	38.2	38.2	100%	6.2	100.00%	3065663	CP189178.1
Vibrio cholerae strain 114Vc08 chromosome, complete genome	Vibrio cholerae	38.2	38.2	100%	6.2	100.00%	4136123	CP189118.1
Vibrio cholerae strain 136Vc02 chromosome 1, complete sequence	Vibrio cholerae	38.2	38.2	100%	6.2	100.00%	3058931	CP188898.1
Vibrio cholerae strain 117Vc06 chromosome, complete genome	Vibrio cholerae	38.2	38.2	100%	6.2	100.00%	4136136	CP189213.1
Vibrio cholerae strain 105Vc06 chromosome 1, complete sequence	Vibrio cholerae	38.2	38.2	100%	6.2	100.00%	3063268	CP189162.1
Vibrio cholerae strain 108Vc06 chromosome 1, complete sequence	Vibrio cholerae	38.2	38.2	100%	6.2	100.00%	3065661	CP189336.1
Vibrio cholerae strain 112Vc04 chromosome, complete genome	Vibrio cholerae	38.2	38.2	100%	6.2	100.00%	4136125	CP189267.1
Vibrio cholerae strain 129Vc02 chromosome 1, complete sequence	Vibrio cholerae	38.2	38.2	100%	6.2	100.00%	3058023	CP188991.1
Vibrio cholerae strain 110Vc03 chromosome 1, complete sequence	Vibrio cholerae	38.2	38.2	100%	6.2	100.00%	3057135	CP189302.1
Vibrio cholerae strain 125Vc04 chromosome 1, complete sequence	Vibrio cholerae	38.2	38.2	100%	6.2	100.00%	3063772	CP189017.1
Vibrio cholerae strain 136Vc03 chromosome 1, complete sequence	Vibrio cholerae	38.2	38.2	100%	6.2	100.00%	3058933	CP188896.1
Vibrio cholerae strain 114Vc02 chromosome 1, complete sequence	Vibrio cholerae	38.2	38.2	100%	6.2	100.00%	3058925	CP189126.1
Vibrio cholerae strain 134Vc06 chromosome 1, complete sequence	Vibrio cholerae	38.2	38.2	100%	6.2	100.00%	3058908	CP188910.1
Vibrio cholerae strain 105Vc08 chromosome 1, complete sequence	Vibrio cholerae	38.2	38.2	100%	6.2	100.00%	3063271	CP189158.1
Vibrio cholerae strain 104Vc03 chromosome 1, complete sequence	Vibrio cholerae	38.2	38.2	100%	6.2	100.00%	3065663	CP189182.1
Vibrio cholerae strain 105Vc03 chromosome 1, complete sequence	Vibrio cholerae	38.2	38.2	100%	6.2	100.00%	3063270	CP189166.1
Vibrio cholerae strain 124Vc02 chromosome 1, complete sequence	Vibrio cholerae	38.2	38.2	100%	6.2	100.00%	3063770	CP189029.1

Job Title

B3 (rpoB)

RID

H67CMPHC014 *Search expires on 11-12 18:27 pm*

Download All

Program

BLASTN Citation

Database

core_nt See details

Query ID

lcl|Query_3791243

Description

None

Molecule type

nucleic acid

Query Length

20

Other reports

Distance tree of results MSA viewer

Filter Results

Organism *only top 20 will appear* exclude

Type common name, binomial, taxid or group name

+ Add organism

Percent Identity to **E value** to **Query Coverage** to

Filter Reset

Sequences producing significant alignments Download Select columns Show 100

select all *100 sequences selected* GenBank Graphics Distance tree of results MSA Viewer

Description	Scientific Name	Max Score	Total Score	Query Cover	E value	Per. Ident	Acc. Len	Accession
Vibrio cholerae strain 105Vc10 chromosome 1, complete sequence	Vibrio cholerae	40.1	40.1	100%	1.6	100.00%	3065653	CP189154.1
Vibrio cholerae strain 138Vc08 chromosome 1, complete sequence	Vibrio cholerae	40.1	40.1	100%	1.6	100.00%	3058933	CP188856.1
Vibrio cholerae strain 111Vc07 chromosome 1, complete sequence	Vibrio cholerae	40.1	40.1	100%	1.6	100.00%	3057147	CP189277.1
Vibrio cholerae strain 123Vc05 chromosome 1, complete sequence	Vibrio cholerae	40.1	40.1	100%	1.6	100.00%	3093912	CP189041.1
Vibrio cholerae strain 134Vc10 chromosome 1, complete sequence	Vibrio cholerae	40.1	40.1	100%	1.6	100.00%	3058911	CP188904.1
Vibrio cholerae strain 135Vc06 chromosome, complete genome	Vibrio cholerae	40.1	72.4	100%	1.6	100.00%	4138010	CP188901.1
Vibrio cholerae strain 104Vc05 chromosome 1, complete sequence	Vibrio cholerae	40.1	40.1	100%	1.6	100.00%	3065663	CP189178.1
Vibrio cholerae strain 114Vc08 chromosome, complete genome	Vibrio cholerae	40.1	72.4	100%	1.6	100.00%	4136123	CP189118.1
Vibrio cholerae strain 136Vc02 chromosome 1, complete sequence	Vibrio cholerae	40.1	40.1	100%	1.6	100.00%	3058931	CP188898.1
Vibrio cholerae strain 117Vc06 chromosome, complete genome	Vibrio cholerae	40.1	72.4	100%	1.6	100.00%	4136136	CP189213.1
Vibrio cholerae strain 101Vc07 chromosome 1, complete sequence	Vibrio cholerae	40.1	40.1	100%	1.6	100.00%	3057149	CP189246.1
Vibrio cholerae strain 117Vc04 chromosome, complete genome	Vibrio cholerae	40.1	72.4	100%	1.6	100.00%	4136118	CP189215.1
Vibrio cholerae strain 105Vc06 chromosome 1, complete sequence	Vibrio cholerae	40.1	40.1	100%	1.6	100.00%	3063268	CP189162.1
Vibrio cholerae strain 108Vc06 chromosome 1, complete sequence	Vibrio cholerae	40.1	40.1	100%	1.6	100.00%	3065661	CP189336.1
Vibrio cholerae strain 112Vc04 chromosome, complete genome	Vibrio cholerae	40.1	72.4	100%	1.6	100.00%	4136125	CP189267.1
Vibrio cholerae strain 129Vc02 chromosome 1, complete sequence	Vibrio cholerae	40.1	40.1	100%	1.6	100.00%	3058023	CP188991.1
Vibrio cholerae strain 110Vc03 chromosome 1, complete sequence	Vibrio cholerae	40.1	40.1	100%	1.6	100.00%	3057135	CP189302.1
Vibrio cholerae strain vc962760 chromosome 1, complete sequence	Vibrio cholerae	40.1	40.1	100%	1.6	100.00%	2985379	CP184803.1
Vibrio cholerae strain 125Vc04 chromosome 1, complete sequence	Vibrio cholerae	40.1	40.1	100%	1.6	100.00%	3063772	CP189017.1
Vibrio cholerae strain 136Vc03 chromosome 1, complete sequence	Vibrio cholerae	40.1	40.1	100%	1.6	100.00%	3058933	CP188896.1
Vibrio cholerae strain 114Vc02 chromosome 1, complete sequence	Vibrio cholerae	40.1	40.1	100%	1.6	100.00%	3058925	CP189126.1
Vibrio cholerae strain 134Vc06 chromosome 1, complete sequence	Vibrio cholerae	40.1	40.1	100%	1.6	100.00%	3058908	CP188910.1
Vibrio cholerae strain 105Vc08 chromosome 1, complete sequence	Vibrio cholerae	40.1	40.1	100%	1.6	100.00%	3063271	CP189158.1

Capture d'écran

Feedback

Supplementary figure S7:

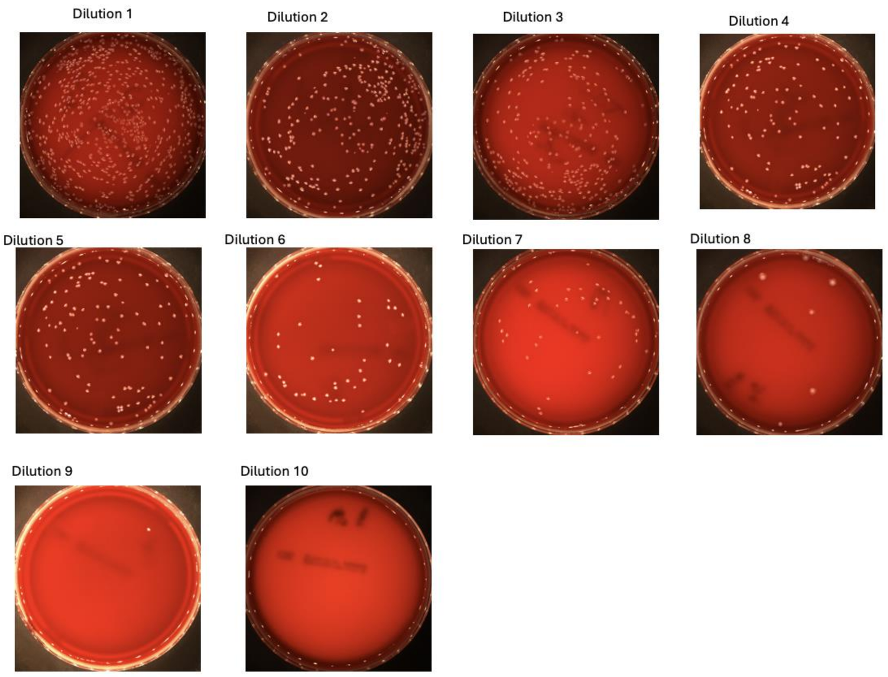

Calibration de l'inoculum *V. cholerae*: Bacterial Suspension 1 Mc Farland / 3*10^8 CFU/mL

As the authors answered all the Reviewers' comments and corrected the manuscript accordingly, they hope that this revised version will be accepted for publication.

Yours Sincerely,

Prof. Elodie TERRER

Corresponding author.

Re: Spectrum02753-25R1 (Isothermal amplification and colorimetric detection of Vibrio cholerae in environmental matrices.)

Dear Prof. Elodie Terrer:

Your manuscript has been accepted, and I am forwarding it to the ASM production staff for publication. Your paper will first be checked to make sure all elements meet the technical requirements. ASM staff will contact you if anything needs to be revised before copyediting and production can begin. Otherwise, you will be notified when your proofs are ready to be viewed.

Data Availability: ASM policy requires that data be available to the public upon online posting of the article, so please verify all links to sequence records, if present, and make sure that each number retrieves the full record of the data. If a new accession number is not linked or a link is broken, provide production staff with the correct URL for the record. If the accession numbers for new data are not publicly accessible before the expected online posting of the article, publication may be delayed; please contact ASM production staff immediately with the expected release date.

Publication Fees: For information on publication fees and which article types have charges, please visit our website. We have partnered with Copyright Clearance Center (CCC) to collect author charges. If fees apply to your paper, you will receive a message from no-reply@copyright.com with further instructions. For questions related to paying charges through RightsLink, please contact CCC at ASM_Support@copyright.com or toll free at +1-877-622-5543. CCC makes every attempt to respond to all emails within 24 hours.

ASM Membership: Corresponding authors may join or renew ASM membership to obtain discounts on publication fees. Need to upgrade your membership level? Please contact Customer Service at Service@asmusa.org.

Embargo Policy: A press release may be issued as soon as the manuscript is posted on the Spectrum Latest Articles webpage. The corresponding author will receive an email with the subject line "ASM Journals Author Services Notification" when the article is available online.

The ASM Journals program strives for constant improvement in our submission and publication process. Please tell us how we can improve your experience by taking this quick Author Survey.

Thank you for submitting your paper to Spectrum.

Sincerely,
Salina Parveen
Editor
Microbiology Spectrum

Reviewer #1 (Comments for the Author):

Thank you for considering all my comments.
